# Second-generation molecular subgrouping of medulloblastoma: an international meta-analysis of Group 3 and Group 4 subtypes

**DOI:** 10.1007/s00401-019-02020-0

**Published:** 2019-05-10

**Authors:** Tanvi Sharma, Edward C. Schwalbe, Daniel Williamson, Martin Sill, Volker Hovestadt, Martin Mynarek, Stefan Rutkowski, Giles W. Robinson, Amar Gajjar, Florence Cavalli, Vijay Ramaswamy, Michael D. Taylor, Janet C. Lindsey, Rebecca M. Hill, Natalie Jäger, Andrey Korshunov, Debbie Hicks, Simon Bailey, Marcel Kool, Lukas Chavez, Paul A. Northcott, Stefan M. Pfister, Steven C. Clifford

**Affiliations:** 1 Hopp Children’s Cancer Centre at National Centre for Tumour Diseases Heidelberg (KiTZ), Heidelberg, Germany; 2 Division of Paediatric Neurooncology, German Cancer Research Center (DKFZ), Heidelberg, Germany; 3 Faculty of Biosciences, Heidelberg University, Heidelberg, Germany; 4 Department of Paediatric Haematology and Oncology, Heidelberg University Hospital, Heidelberg, Germany; 5 Wolfson Childhood Cancer Research Centre, Northern Institute for Cancer Research, Newcastle University, Newcastle upon Tyne, UK; 6 Department of Applied Sciences, Northumbria University, Newcastle upon Tyne, UK; 7 Department of Pathology and Center for Cancer Research, Massachusetts General Hospital and Harvard Medical School, Boston, MA 02114 USA; 8 Broad Institute of Harvard and MIT, Cambridge, MA 02142 USA; 9 Department of Pediatric Hematology and Oncology, Center for Obstetrics and Pediatrics, Universitatsklinikum Hamburg-Eppendorf, Hamburg, Germany; 10 Department of Oncology, St. Jude Children’s Research Hospital, Memphis, TN 38105 USA; 11 Programme in Developmental and Stem Cell Biology, The Hospital for Sick Children, Toronto, ON Canada; 12 Division of Haematology/Oncology, Department of Pediatrics, The Hospital for Sick Children, 555 University Ave, Toronto, ON M5G 1X8 Canada; 13 Department of Laboratory Medicine and Pathobiology, University of Toronto, Toronto, ON Canada; 14 Division of Clinical Cooperation Unit Neuropathology, German Cancer Research Center (DKFZ), Heidelberg, Germany; 15 Department of Medicine, University of California, San Diego, USA; 16 Department of Developmental Neurobiology, St. Jude Children’s Research Hospital, Memphis, TN 38105 USA

**Keywords:** Medulloblastoma, Subtypes, Meta-analysis, Biomarkers, Methylation

## Abstract

In 2012, an international consensus paper reported that medulloblastoma comprises four molecular subgroups (WNT, SHH, Group 3, and Group 4), each associated with distinct genomic features and clinical behavior. Independently, multiple recent reports have defined further intra-subgroup heterogeneity in the form of biologically and clinically relevant subtypes. However, owing to differences in patient cohorts and analytical methods, estimates of subtype number and definition have been inconsistent, especially within Group 3 and Group 4. Herein, we aimed to reconcile the definition of Group 3/Group 4 MB subtypes through the analysis of a series of 1501 medulloblastomas with DNA-methylation profiling data, including 852 with matched transcriptome data. Using multiple complementary bioinformatic approaches, we compared the concordance of subtype calls between published cohorts and analytical methods, including assessments of class-definition confidence and reproducibility. While the lowest complexity solutions continued to support the original consensus subgroups of Group 3 and Group 4, our analysis most strongly supported a definition comprising eight robust Group 3/Group 4 subtypes (types I–VIII). Subtype II was consistently identified across all component studies, while all others were supported by multiple class-definition methods. Regardless of analytical technique, increasing cohort size did not further increase the number of identified Group 3/Group 4 subtypes. Summarizing the molecular and clinico-pathological features of these eight subtypes indicated enrichment of specific driver gene alterations and cytogenetic events amongst subtypes, and identified highly disparate survival outcomes, further supporting their biological and clinical relevance. Collectively, this study provides continued support for consensus Groups 3 and 4 while enabling robust derivation of, and categorical accounting for, the extensive intertumoral heterogeneity within Groups 3 and 4, revealed by recent high-resolution subclassification approaches. Furthermore, these findings provide a basis for application of emerging methods (e.g., proteomics/single-cell approaches) which may additionally inform medulloblastoma subclassification. Outputs from this study will help shape definition of the next generation of medulloblastoma clinical protocols and facilitate the application of enhanced molecularly guided risk stratification to improve outcomes and quality of life for patients and their families.

**Electronic supplementary material:**

The online version of this article (10.1007/s00401-019-02020-0) contains supplementary material, which is available to authorized users.

## Introduction

Medulloblastoma (MB; World Health Organization grade IV) is a highly malignant brain tumor mainly occurring in childhood [16]. In 2005, it was reported that beta-catenin (*CTNNB1*) mutations defined a group of MBs with activated WNT/wingless signaling, which was associated with favorable outcome [5, 8, 24]. Subsequently, multiple independent unsupervised gene expression microarray studies conducted on medium-sized MB cohorts (typically *n* < 200) described between 4 and 6 molecular subgroups [4, 14, 15, 30]. In 2012, an international consensus on MB subgroups was reached amongst the pediatric neuro-oncology community, reporting four distinct MB subgroups: WNT, SHH, Group 3 (Grp3), and Group 4 (Grp4) [24, 29]. Since publication of this consensus, the biological and clinical relevance of MB subgroups has been extensively reported, including methods for robustly assigning clinical samples to subgroups based on either transcriptomic [22] or methylomic signatures [12, 25, 27]. Together, these advances recently culminated in the recognition of MB subgroups as part of the WHO Classification of CNS tumors [17, 24], which currently recognizes four molecular variants of the disease (WNT, SHH-*TP53*^wild type^, SHH-*TP53*^mut^, and non-WNT/non-SHH). The non-WNT/non-SHH subgroup encompasses the Grp3 and Grp4 consensus molecular variants of medulloblastoma.

WNT MB patients aged under 16 typically have a favourable prognosis (> 90% survival); the prognosis for adult WNT MB patients is less clear [5, 7, 8, 11]. SHH MB patients exhibit heterogeneous outcomes that are associated with patient age at diagnosis, histopathology, and specific underlying genetics (i.e., *TP53* mutation status) [11, 26, 32]. The defining biology of Grp3 and Grp4 MB remains less clear. *MYC* amplification is a hallmark feature of Grp3 MB, occurring in ~ 15–20% of patients and associated with a poor clinical outcome. Chromatin modifier alterations are common in Grp4 MB, collectively contributing to ~ 30–40% of patients. Whole-chromosome abnormalities alongside isochromosome 17q are common in both Grp3 and Grp4 MB [21]. Activated expression of *GFI1* or *GFI1B* transcription factors through a structural variant-dependent mechanism termed ‘enhancer hijacking’ likewise contributes to subsets of Grp3 and Grp4 tumors [20].

Grp3 and Grp4 represent ~ 65% of all MB cases and have heterogeneous clinical characteristics and survival outcomes. In part, these are associated with known high-risk disease factors (patients < 3 years of age at diagnosis, *MYC* amplification, large cell/anaplastic (LCA) histology, and metastatic disease) [23]. However, substantial numbers of Grp3/Grp4 patients relapse in the absence of these risk factors [11, 29]. A more refined understanding of their molecular heterogeneity is urgently needed for improved disease subclassification, stratification of current treatments, and the development of novel, subgroup-directed therapies.

**Fig. 1:**
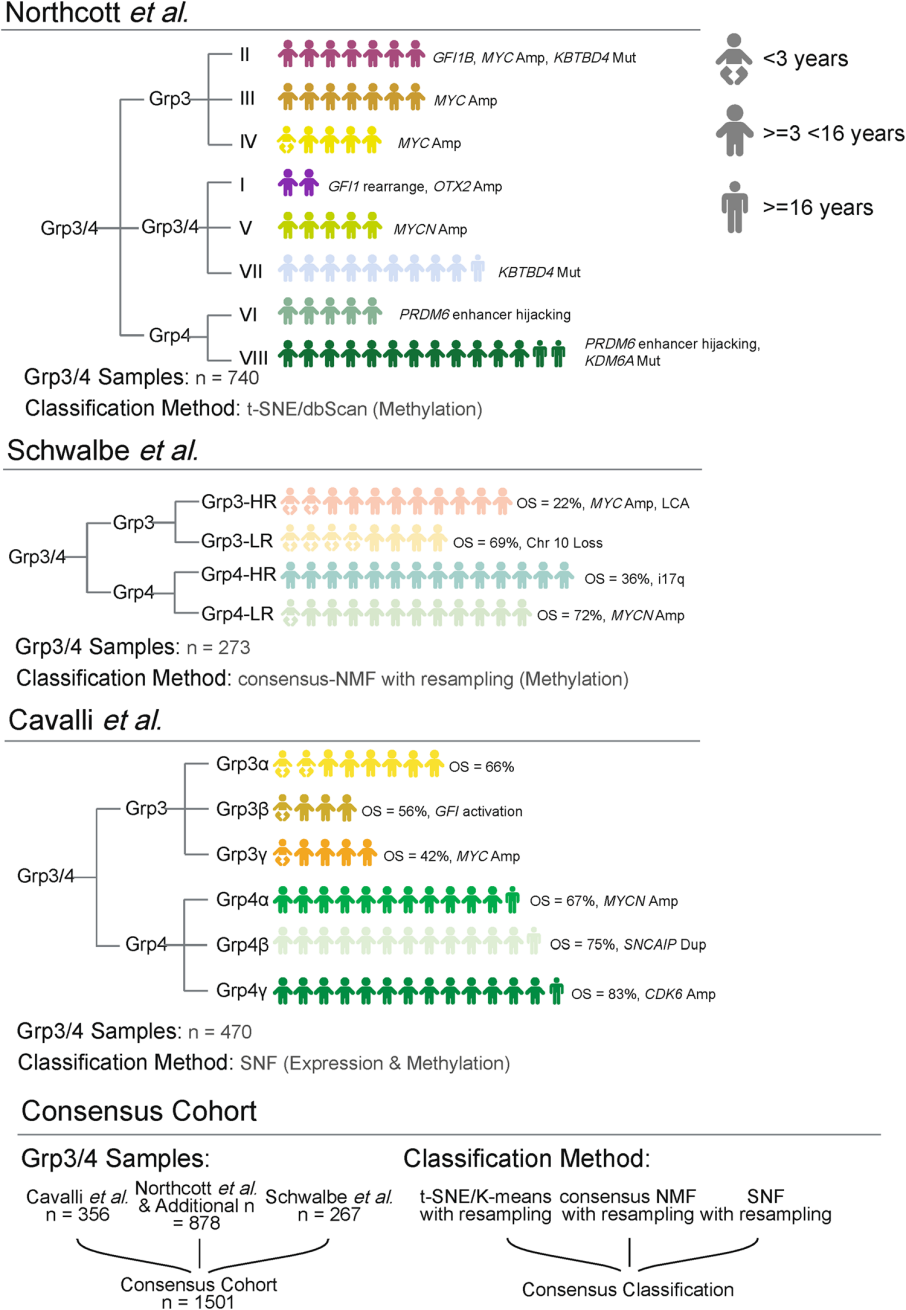
Summary of ‘second-generation’ medulloblastoma subgrouping of Grp3/4 medulloblastoma. **a** For each component study, the reported subtypes of Grp3/4 are shown, alongside incidence, age where possible, methodology, and major study findings. For each individual study, the most frequent subtype was scaled to 14 human figures; less frequently occurring subtypes were scaled from there. *LR* low risk, *HR* high risk. The analytical approach employed in a unified Grp3/4 cohort in this study is outlined in **b**. *t-SNE* t-stochastic neighbor embedding, *NMF* non-negative matrix factorization, *SNF* similarity network fusion

In 2017, three independent studies investigated the molecular and clinical features of MB subgroups in larger cohorts and at higher genomic resolution than had previously been reported [3, 18, 26] (Fig. 1).

Using non-negative matrix factorization (NMF) to define metagenes, composite measures that reflect the methylation status of many molecular features to describe the major biological effects within a data set, Schwalbe et al. [26] reported seven molecular subtypes of MB through unsupervised class discovery based on 428 MBs profiled by DNA-methylation array [6]. While WNT remained a single entity, SHH split into two age-dependent subtypes. Likewise, Grp3 and Grp4 each split into two high- and low-risk groups, with clear evidence for shared biology between them.

Cavalli et al. performed an integrative analysis of 763 MBs profiled by both gene expression and DNA-methylation array using a technique called similarity network fusion (SNF). This technique constructs networks for each discrete data type (e.g., DNA methylation and gene expression) and then fuses them to give an overarching view of a disease. In their analysis, they identified 12 molecular subtypes: the four consensus subgroups were analyzed independently of each other; WNT was split into two age-dependent subtypes, SHH into four subtypes, and Grp3 and Grp4 were each split into three subtypes (alpha, beta, and gamma) [3].

Northcott et al. applied *t*-Distributed Stochastic Neighbor Embedding (*t*-SNE), a dimension reduction technique, followed by clustering using the DBSCAN (density-based clustering of applications with noise) algorithm. *t*-SNE reduces the variation within a complex data set to typically 2 or 3 dimensions. While it has similarities to the more familiar principal component analysis (PCA), *t*-SNE is more effective at maintaining local differences between samples while preserving larger scale, global differences between different subgroups. DBSCAN is a clustering algorithm that groups samples based on their proximity (or density). This combined *t*-SNE/DBSCAN approach was applied to a series of 1256 MBs profiled by DNA-methylation array [18]. Class discovery was undertaken by analyzing Grp3 and Grp4 subgroups together (*n* = 740), identifying eight subtypes (I–VIII).

It may be hypothesized that the differing number of reported subtypes between studies, particularly within Grp3 and Grp4, is a result of different analytical approaches, parameter choice and cohort composition. There is now an urgent need to reach an agreement, which resolves the apparent inconsistencies in subtype numbers between studies, to provide the requisite clarity to provide a consistent basis for biological studies and enable subsequent planning of future MB clinical trials.

Herein, we aimed to characterize the number and nature of Grp3 and Grp4 MB subtypes in an unbiased way, in a genomically characterized cohort of 1501 such MBs. We applied the same analytical techniques and approaches used in each of the previously described studies, in conjunction with classification confidence measures, to objectively identify robust and reproducible concordant subtypes within Grp3 and Grp4. We describe their major clinico-pathological and molecular features, their relationship to the original consensus Grp3 and Grp4 definitions and proffer a routine classification tool to prospectively assign MB samples to these eight concordant subtypes.

## Materials and methods

### Sample classification and pre-selection

We combined data for all MB samples from the three published cohorts [3, 18, 26], and added methylation profiles from an additional 153 tumors. All samples were classified using the MNP2.0 v11b4 Random Forest classifier [2]; only samples classified as Grp3 and Grp4 with a calibrated prediction score of 0.90 were selected for further analysis. The excluded samples are characterized in Supplementary Tables 1 and 2 and non-MB predictions are shown in supplementary Fig. 2. Grp3 and Grp4 sample profiles were considered together; no a priori assumptions about the absolute separation or otherwise of Grp3 and Grp4 samples were made.

**Fig. 2:**
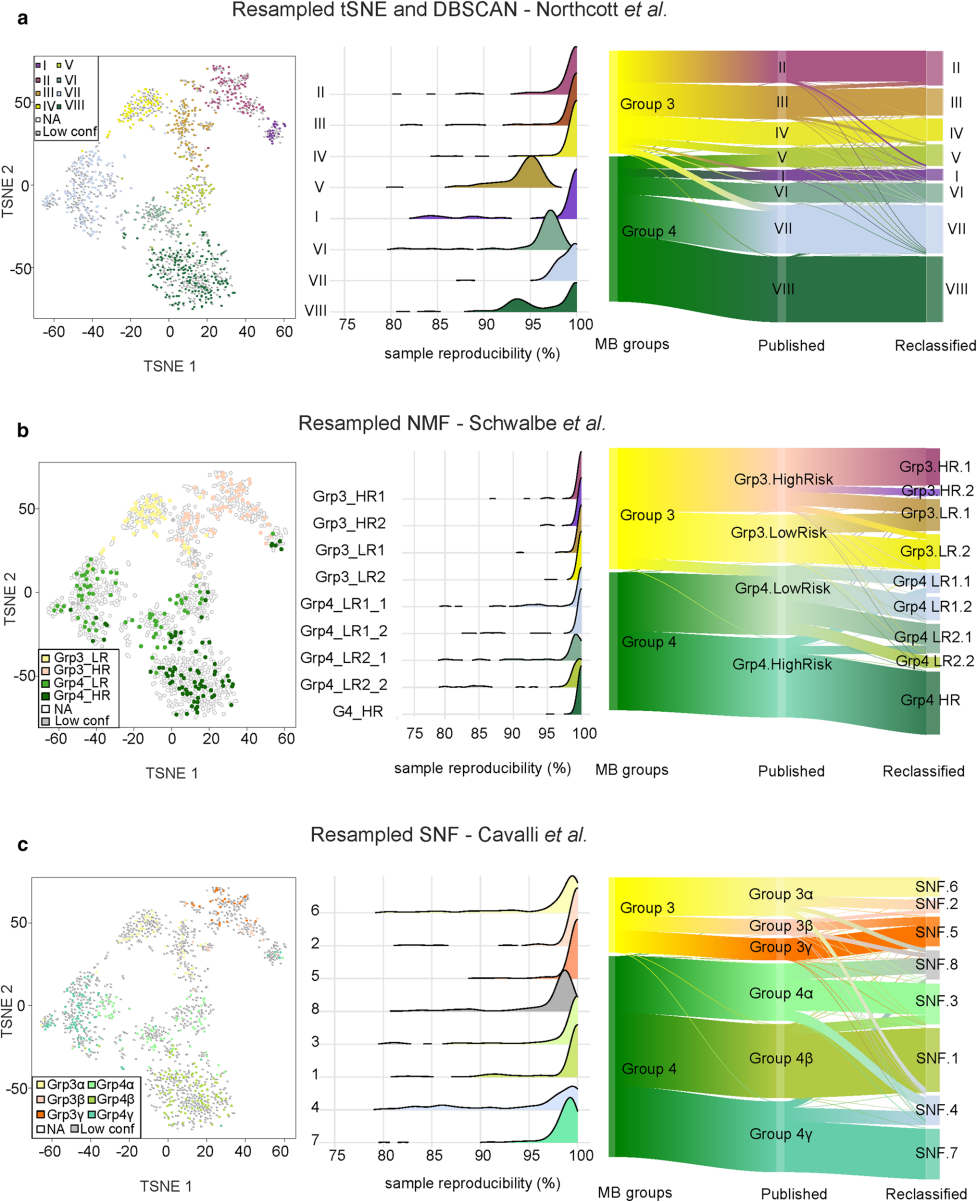
Consensus clustering identifies substructure within Grp3/4 medulloblastoma. Each row shows the results of applying t-SNE (**a**), NMF (**b**), and SNF (**c**), respectively, to the Grp3/4 cohort (*n* = 1501). For each row, a common *t*-SNE visualization is shown in the first panel, which depicts study-specific samples with their original study-specific subtype designation. The samples not included in a study were annotated as empty ‘NA’ in the t-SNEs (marked as empty circles). The samples that could not be assigned to a concordant subtype for a corresponding technique were annotated as ‘Low conf’ (marked in grey). In the second column, the reproducibility of the identified sample subtype is shown. Density plots show the distribution of sample reproducibility from consensus clustering by subtype for the selected number of clusters for each approach—t-SNE, NMF, and SNF. In the third column, Sankey plots demonstrate the relationship of the published subtypes to the assigned subtypes when clustered as part of a larger cohort. **c** The SNF results are from analysis of DNA-methylation data only

Initial data import, normalization, and quality control of the methylation arrays were performed using the R package ‘minfi’, as previously described [18]. All duplicated MB samples (*n* = 80) were removed [2, 18]. Copy-number changes for the resultant unique, non-duplicated cohort (*n* = 1501), were identified using the conumee package (v 1.9.0), as previously described [18, 26].

Analytical techniques used in the component studies of this combined Grp3 and Grp4 cohort (NMF, SNF, and t-SNE/DBSCAN) were performed as previously described [3, 18, 26], but with the addition of a common consensus clustering approach to assess cluster stability and reliability. Briefly, we carried out iterative resampling of the data sets, randomly selecting 80% of the data set at each iteration. At each iteration, the identified clusters were mapped back to ‘gold-standard’ clusters identified using the entire data set and cluster reproducibility was assessed using Cohen’s kappa. Individual samples were classified as ‘not-assignable’ if their frequency of assignment to the modal cluster fell below 80% [26]. For each technique, our overarching aim was to identify a maximal number of robustly assigned subtypes (supplementary Fig. 4).

### t-SNE/dbSCAN identification of subtypes

For unsupervised *t*-SNE analysis of the 1501 MB samples, we selected the 15,335 most variably methylated probes (weighted standard deviation > 0.25). We defined a distance measure (‘*1*—*the weighted Pearson correlation coefficient*’) and used this to calculate pairwise sample distances, using the wtd.cors function of the weights R package (v.0.85), which gives more variable probes greater influence. The resulting distance matrix was used to perform the *t*-SNE analysis (Rtsne package v.0.13), using previously described parameters [18]. The clusters were identified using the DBSCAN algorithm as implemented in the dbscan package v.0.9-7. Samples not assigned to any cluster were iteratively merged to their nearest cluster as previously described [18]. For the consensus application of this approach, we performed 2500 resamplings, each selecting 80% of the data set, and, at each iteration, calculated *t*-SNE coordinates for a range of dimensions (2–10) and assigned 2–10 clusters using *k*-means, since this allows the specification of a fixed number of clusters.

### NMF consensus clustering

Consensus clustering of the cohort was performed as previously described [26]. Briefly, using 250 resamplings of 80% of the data set (as in the component study), we performed non-negative matrix factorization to identify from 2 to 10 metagenes within the data set, and projected them back onto the entire cohort. These metagenes were then clustered into 2–10 groups using *k*-means. Clusters were mapped against gold-standard clusters identified using the entire data set; cluster and sample stability were assessed as for t-SNE consensus analysis. Optimal combinations of subtype and metagene number were identified as previously reported [26]. Five optimal subtypes were identified using this approach. We repeated NMF consensus clustering for each of these five defined subtypes in isolation, which we termed ‘second-order’ NMF, to examine whether any additional subtypes could be identified.

### Similarity network fusion

We applied Similarity Network Fusion (SNF) as employed by Cavalli et al. [3], on the entire DNA-methylation array cohort, as well as on three subcohorts with paired DNA methylation/gene expression data. These cohorts were unsuitable to assess in combination due to differing transcriptomic platforms (Affymetrix Exon array data (*n* = 763) [3], Affymetrix 3′IVT expression arrays (*n* = 250) [18], and RNA-seq data (*n* = 131) [26].

For the methylation array only cohort (*n* = 1501), we performed consensus SNF clustering using the SNFtool package v 2.2, different parameters for *k* (10, 20, 30, and 40) and varying numbers of *k*-means clusters (2–10). Cluster and sample stabilities were assessed as per *t*-SNE analysis. For the paired methylome/transcriptome analyses, we customized parameters specifically for Grp3 and Grp4, as described by Cavalli et al. (*k* = 20, *α* = 0.5, *T* = 10, *C* = 2) [3]. For transcriptome data, the 10% most variably expressed genes were preselected; for methylation data, the 10% most variably methylated CpG loci were selected for creating patient similarity matrices. The fused output similarity matrices with display clusters were then visualized.

### Assignment of concordant subtypes

The technique-dependent subtype assignments were compared by creating Sankey plots (also known as alluvial plots) using the googleVis package and through plotting study-specific subtype allocations onto a common t-SNE visualization of all 1501 samples. Concordance between technique-dependent subtypes were identified and retained as defined subtypes. Where there was potential ambiguity between technique-dependent subtype assignments or intra-technique subtype number choice, additional analyses were performed to test for clinico-pathological and cytogenetic differences between subtypes; subtype assignments which showed clear clinico-pathological and/or cytogenetic differences were favored over assignments where subtype-splits demonstrated few or no differences.

After identifying final consensus technique-dependent subtype assignments, and their mapping between techniques, we assigned samples to a specific subtype if there was concordance of assignment in ≥2 out of 3 studies; samples not meeting this criterion were classed as ‘low-confidence’. These concordant subtypes were once again visualized using *t*-SNE.

### CNV heatmaps and oncoplots

After assignment to concordant subtypes, copy-number data generated with conumee [13] were hierarchically clustered within each subtype by their distance measure. Samples with poor-quality copy-number estimates (defined by a noise parameter greater than 2.5) were excluded. Chi squared tests were used to identify subtypes with significantly enriched CNVs, as previously described [18, 26]. An oncoplot showing focal copy-number amplifications for *MYC, MYCN, OTX2,* and *CDK6* was generated, as previously described [9]; significant enrichments for focal amplifications were assessed using Chi squared tests.

### Survival/clinico-pathology analysis

We identified the clinico-pathological and survival characteristics of the newly characterized subtypes of Grp3/Grp4. We used ANOVA to identify differences in patient age and plotted their age distribution as density plots. Chi squared tests were used to identify subtype association with sex, histopathology, and metastatic stage. Using Kaplan–Meier analysis, we characterized progression free and overall survival for the entire cohort, as well as restricting analyses to patients aged ≥ 5 years at diagnosis, to remove potentially confounding treatment differences in infant patients [23].

### Grp3 and Grp4 classifier (https://www.molecularneuropathology.org/mnp/classifier/7)

To predict subtypes of Grp3 and Grp4 samples, a Random Forest (RF) [28] classifier with an accompanying score calibration model was trained as previously reported [2]. Briefly, batch adjustment for FFPE/fresh-frozen derivatives and adjustments for 450 k/EPIC array methylated and unmethylated signals was performed by a two-factor linear model on their log_2_ intensities. Feature selection was applied to the 50,000 CpG loci with the highest standard deviation and 10,000 CpG loci with the highest RF permutation variable importance. Using a multinomial logistic regression with a ridge-penalization term, RF score calibration was implemented to ensure well-calibrated class probability estimates (defined by low Brier score). A threefold nested cross validation was then performed to validate the classifier on the data set used in its creation.

## Results

### Identification of eight molecular subtypes

We undertook an integrative analysis of Grp3 and Grp4 subtypes using combined cohorts from three published component studies (supplementary Fig. 1) [3, 18, 26], whose respective major findings are summarized in Fig. 1. Quality control checks revealed no biases attributable to sample type (i.e., fresh frozen vs formalin-fixed, paraffin embedded), array type (450 k vs 850 k), or source study/institution (supplementary Fig. 3). A subset of samples were removed due to low methylation classifier prediction scores < 0.9 (*n* = 69; supplementary Table 1) [2] or due to shared genotypes between studies (i.e., duplicated samples; *n* = 80). Additionally, 27 samples were removed due to a ‘non-MB’ prediction by the methylation classifier (supplementary Table 2, supplementary Fig. 2). The resultant combined DNA-methylation array cohort represented 1501 Grp3 and Grp4 MBs and their demographics are summarized in Table 1.

**Table 1:** Clinico-pathological and molecular features of component studies

	Cavalli et al.	Northcott et al.	Schwalbe et al.	*p*
Cohort size	356	878	267	NA
Age at diagnosis (years)				
Median (min–max)	8.0 (1–49.6)	7.3 (1.5–28)	6.4 (0.5–16)	< 0.0001
Age available	337	366	267	
Age unknown	19	512	0	
Sex				
M	239	265	185	0.81
F	96	106	82	
Sex unknown	21	507	0	
M:F ratio	2.5:1	2.5:1	2.3:1	
Molecular subgroup				
Group 3	94	336	108	< 0.0001
Group 4	262	542	159	
Group 3:Group 4 ratio	0.36:1	0.62:1	0.68:1	
Histology				
CLA	200	117	204	0.0002
DN	30	6	9	
LCA	27	5	33	
Unknown	99	750	21	
Survival information				
PFS	0	291	259	0.00031
No PFS available	356	587	8	
Median PFS (years)	NA	6.5	5.3	
OS	283	291	263	0.89
No OS available	73	587	4	
Median OS (years)	5.0	5.0	5.2	
*MYC* amplification				
Amplified	10	57	18	0.020
Not amplified	342	815	249	
Unknown	4	6	0	
*MYCN* amplification				
Amplified	9	41	8	0.18
Not amplified	343	831	259	
Unknown	4	6	0	

*p* values are given from ANOVA (age at diagnosis) and from Chi squared tests (all other *p* values)

Consensus clustering was applied to the entire data set to identify the maximal number of robustly defined subtypes beyond the two consensus subgroups 3 and 4. We independently employed each of the sample clustering techniques used in the three component studies. Using *t*-SNE/*k*-means [18], cluster reproducibility reached a maximum at five clusters, and dropped substantially when moving beyond eight clusters (Fig. 2a, supplementary Fig. 4). The eight identified clusters were highly congruent with the eight subtypes previously reported by Northcott et al. and were resolved by bifurcations of three of the five clusters which were most reproducibly identified [18] (Fig. 2, supplementary Fig. 4).

Using NMF/*k*-means [26], a first analysis identified an optimal solution of five subtypes, defined by six metagenes (Fig. 2, supplementary Fig. 4). Second-order NMF consensus clustering within each of these five groups identified additional heterogeneity, resulting in a total of nine robust subtypes (Fig. 2b, supplementary Fig. 4); these demonstrated clear associations with the originally published subtypes [26] (Fig. 2b).

SNF consensus clustering [3] of DNA-methylation data supported six clusters, as originally reported, but also offered support for the derivation of eight stable clusters (Fig. 2c, supplementary Fig. 4). Some clusters contained both Grp3 and Grp4 constituents, consistent with the subtypes identified by t-SNE or NMF above.

In their original report, Cavalli et al. reported the application of SNF clustering to paired DNA methylation and expression data. When applied to the Cavalli et al. data set, we retrieved the subtypes as published (data not shown). However, we could also stratify this data set into eight subtypes when using SNF (supplementary Fig. 5). For the subset of the Northcott et al. data set for which both data types were available, eight subtypes were revealed when using paired DNA methylation/transcriptome data (supplementary Fig. 6). For a subset of the Schwalbe et al. data set with paired data, SNF resulted in improved stratification between Grp3 and Grp4 subtypes, recapitulating the originally published subdivisions with fewer samples (supplementary Fig. 6). Collectively, these results indicate that one of the analytical strengths of SNF is its ability to identify MB subtypes with fewer samples [18].

### Definition of subtypes

**Fig. 3:**
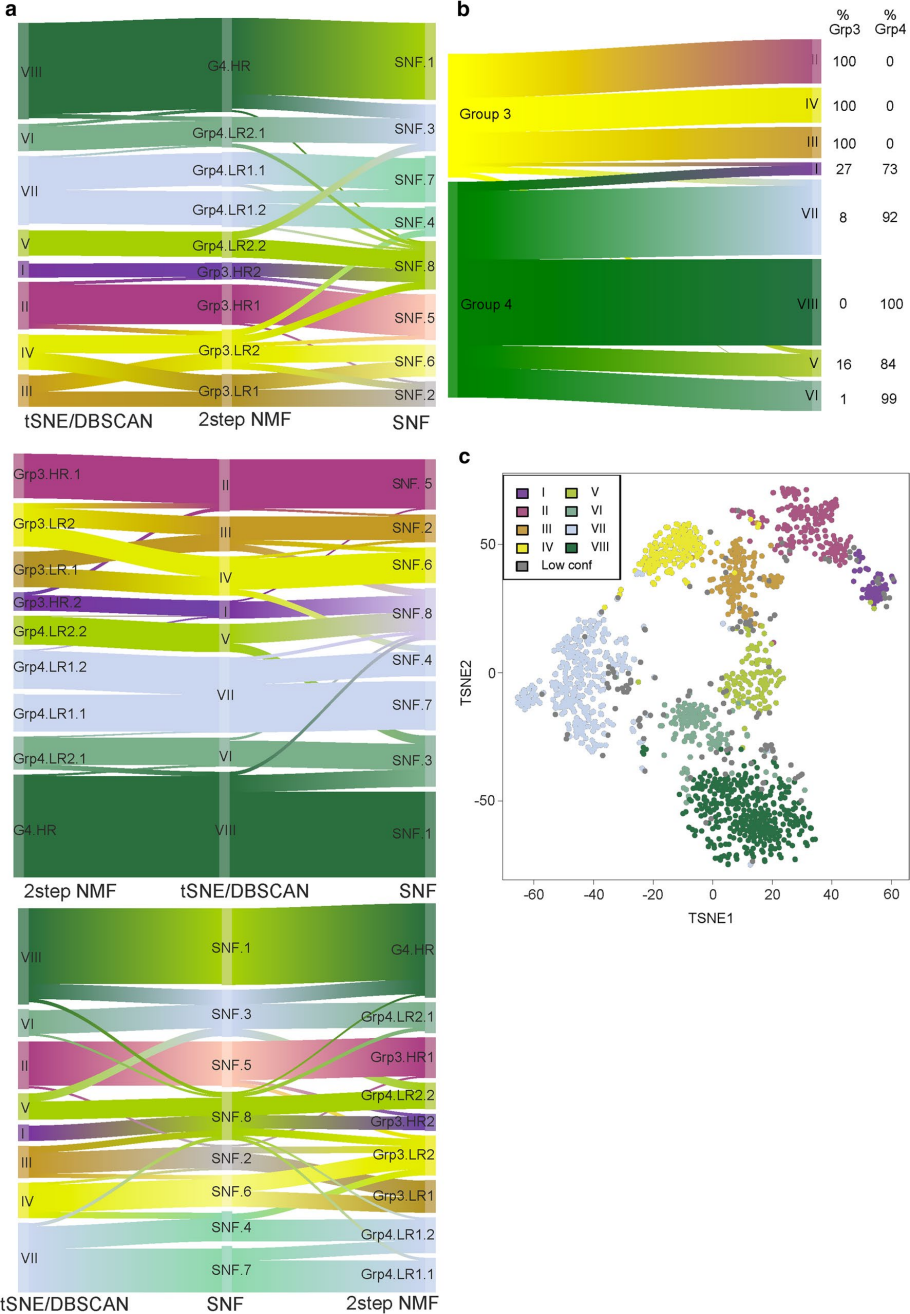
Inter-technique comparisons identify eight subtypes within Grp3/4 medulloblastoma. **a** Sankey plots show the relationship of subtype calls from each technique to those from other techniques. The same information is repeated three times for the Sankey plot; each plot shows a different technique in the center, to enable inter-technique relationships to be identified. **b** Sankey plot shows relationship between subtype calls and Grp 3 and 4 subgroup membership. **c** t-SNE visualizing subtype calls of 1501 Grp3/4 samples at weighted standard deviation > 0.25 with 15,335 most variable probes. Samples assigned to the same subtype from ≥ 2 techniques were assigned to that subtype. Unassignable samples were annotated as ‘Low conf’. *DBSCAN* density-based spatial clustering algorithm, used to identify subtypes after t-SNE dimension reduction. Two-step NMF refers to the two stages of NMF analysis; five robust subtypes were identified in the first stage. In the second stage, NMF consensus clustering was applied to each of the five subtypes in isolation

After defining subtypes by NMF, t-SNE, and SNF, we assessed subtype concordance between techniques. Subtype Grp3.HR.1/II/SNF.5 showed near 1:1 mapping across all three techniques (Fig. 3b, supplementary Table 3). Other subtypes showed agreement across 2/3 techniques. For example, Grp3.HR.2 (NMF) mapped to I (t-SNE); however, this subtype was not identified by SNF. Subtype Grp4.LR2.2 (NMF) mapped to subtype V (t-SNE), which mapped to SNF subtypes 3 and 8. G4.HR (NMF) and VIII (t-SNE) mapped to SNF subtypes 1 and 3. Similarly, subtype 3 (SNF) mapped to Grp4.LR2.1 (NMF) and subtype VI (t-SNE), which themselves showed close concordance.

Subtypes III and IV (t-SNE) were concordant with SNF subtypes 2 and 6, respectively; however, NMF subtypes Grp3.LR1 and Grp3.LR2 mapped evenly between them. Of note, t-SNE subtype VII could be further split into two subtypes by NMF and SNF; however, these splits were not congruent (supplementary Figs. 7, 8). Taken together, we identified five subtypes supported by ≥ 2 techniques (supplementary Table 3), with three additional subtypes requiring further investigation.

To resolve the discrepancy between subtypes III, IV (t-SNE)/2,6 (SNF) and their associated NMF assignments, as well as the split of t-SNE subtype VII into two further subtypes by SNF or NMF, we investigated their clinico-pathological, molecular, and cytogenetic correlates. For subtypes III and IV (t-SNE)/2,6 (SNF), there was notable clinico-biological support for their distinction by virtue of distinct survival and cytogenetic differences between III and IV that were not apparent when comparing Grp3.LR1 and Grp3.LR2 (supplementary Fig. 9). Thus, we adopted the III/IV, 2,6 subtype distinction as true concordant subtypes. For the putative split of t-SNE subtype VII, there was clinico-biological support for the adoption of the NMF-derived subtypes (supplementary Fig. 7) in preference to the SNF subtypes 7 and 4; Grp4.LR1.1 had an older age of incidence, a worse survival and was hypomethylated compared to Grp4.LR1.2; moreover, Grp4LR1.2 was enriched for gains of chr7p and chr18 and loss of chr8p, among others. The split identified by SNF did not have such striking correlations with clinico-pathological features (supplementary Fig. 8); thus, we did not opt for this additional split. Due to the differing constitution of the split of t-SNE subtype VII by NMF or SNF clustering, we elected not to adopt this additional subdivision of VII into two formal subtypes.

After defining the eight subtypes, we designated them I–VIII, in accordance with the eight subtypes described by Northcott et al., and recognized that there was some evidence for two variants of VII (VII-A, VII-B; Fig. 3, supplementary Fig. 7). Using the final subtype mappings across techniques, we assessed inter-technique sample assignments to define final calls for all samples. Overall, subtype calls from NMF and *t*-SNE-based approaches showed the highest concordance [1228/1501 (82%) tumors]. SNF subtypes demonstrated somewhat less concordance with their *t*-SNE-derived counterparts [1034/1501 (69%) concordant calls], and NMF-derived counterparts [1028/1501 (68%) concordant calls] using methylation data only. Their relationship to the consensus Grp3/4 subtypes is shown in Fig. 3b.

### Grp 3/4 subtypes have distinct cytogenetics

**Fig. 4:**
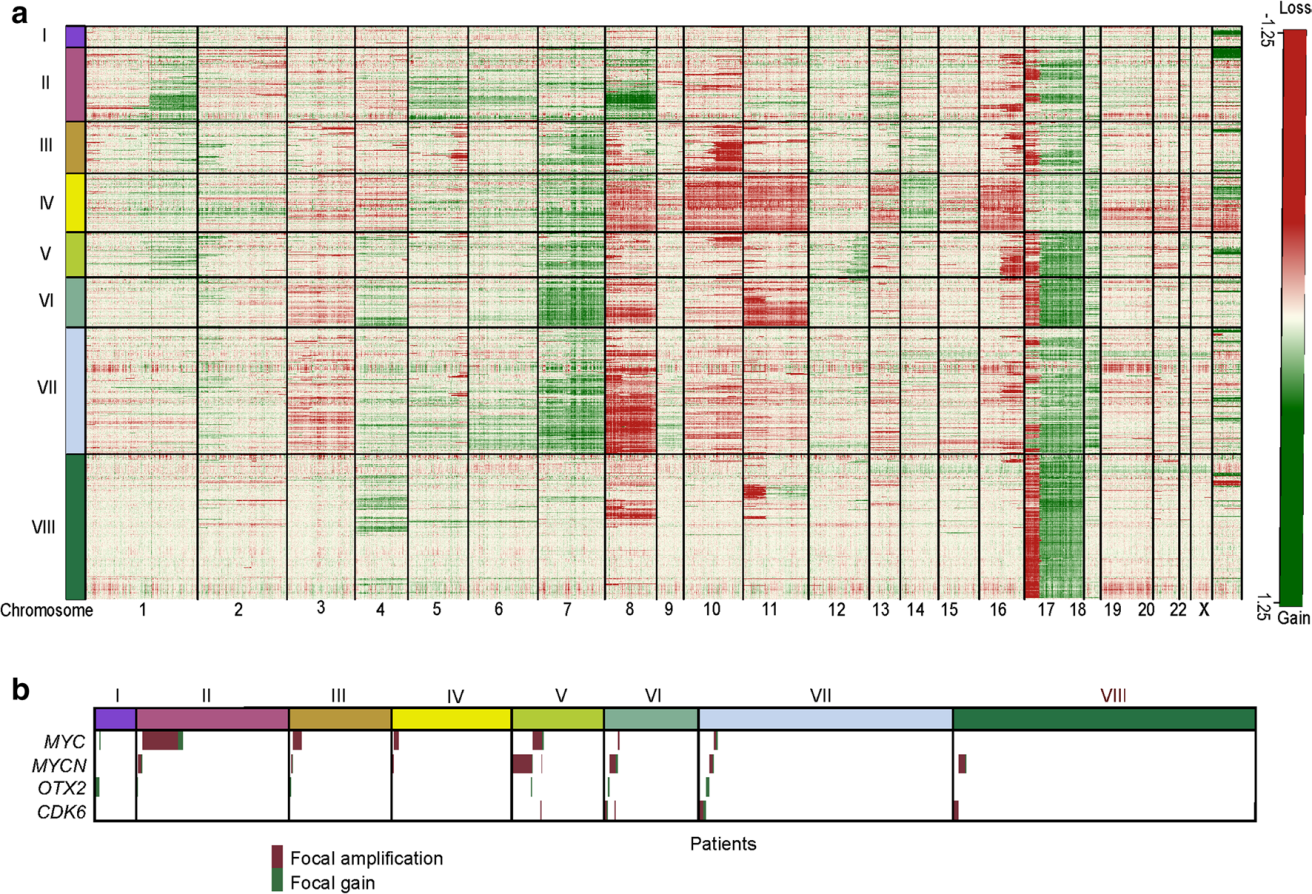
Subtypes have distinct copy-number profiles and significant enrichments of oncogene amplification. **a** CNV heatmap generated from raw conumee calls on methylation data for each subtype across all chromosomes revealed subtype-specific cytogenetic aberrations. Gains are shown in green, losses in red. **b** oncoplot shown summarizes the type and incidence of aberrations for *MYC*, *MYCN*, *OTX2*, and *CDK6*. Focal amplifications are shown in burgundy, focal gains are shown green

Investigating copy-number variants (CNVs) derived from the DNA-methylation array data set [13] revealed cytogenetic events highly enriched in specific subtypes that lend further support to their definition (Fig. 4a). Subtype I was notable for its generally balanced genome.

Subtype II was distinguished by significant (for arm-level/whole chromosomal alterations, all *p* < 0.0001, unless otherwise stated) enrichment of chr8 gain [78/173(45%)], chr13q gain [34/173 (20%)], and chr1q gain [63/173 (36%)]. Subtype III was significantly enriched for chr8p loss [30/115 (26%)] and chr10q loss [68/115 (59%)]. Subtype IV was characterized by significant losses of chr8 [69/139 (50%)], chr10 [78/139 (56%)], chr11 [89/139 (64%)], and chr13 [45/139 (32%)]. Chr13 loss has previously been previously reported as a marker of good prognosis in Grp3 and Grp4 [26].

Subtype V had mixed patterns of aberrations: 58/105 (55%) exhibited i17q, while 52/105 (50%) had chr16q loss. Subtypes VI and VII demonstrated significant gain of chr7 [97/115 (84%) and 161/297 (54%), respectively] and loss of chr8 [39/115 (34%) and 166/297 (56%), respectively]. Subtype VIII exhibited a relatively balanced genome with a majority harboring i17q [279/343 (81%)]. Loss of chrX was frequently observed in subtypes IV, V, VI, VII, and VIII; however, the most predominant chrX loss was seen in subtype VIII, where 35% (21/60) of assessable females showed chrX loss (*p *= 0.11).

The oncoplot shown in Fig. 4b summarizes the type and incidence of focal CNVs affecting commonly altered MB driver genes. Subtype I was significantly enriched for *OTX2* amplification (p < 0.0001), observed in 12% (6/50) of patients (Fig. 4b). Subtype II was significantly enriched [23% (42/179)] for *MYC* amplification (*p *< 0.0001), which was also common in subtype III [8% (10/120)]. Subtype V was significantly enriched for both *MYC* [11/108 (10%), *p* < 0.0001] and *MYCN* [23/108 (21%), *p* < 0.0001] amplifications. Other mutational correlates were as described by Northcott et al. (data not shown).

In summary, subtype-specific enrichments of specific cytogenetic and focal CNVs outlined above further supports the subdivision of Grp3 and Grp4 MB into eight distinct molecular subtypes.

### Clinico-pathological and molecular correlates

**Fig. 5:**
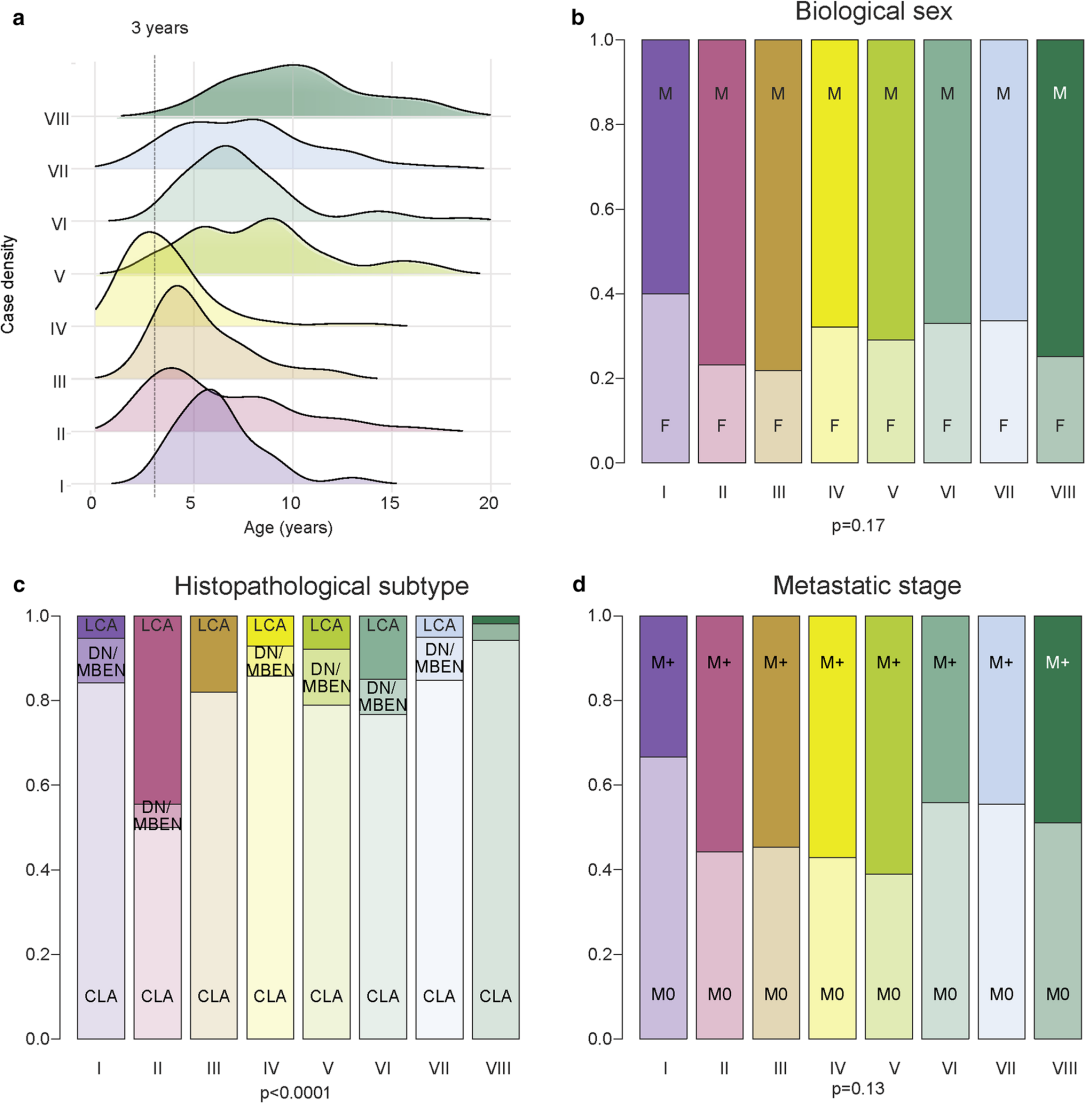
Clinico-pathological associations of Grp3/4 subtypes. **a** Subtypes show distinct age distributions. Density plot shows age distribution for each subtype. **b**–**d** Barplots show incidence of major clinico-pathological features (sex, histopathology, and metastatic stage, respectively) across subtypes. **b** No difference in distribution of biological sex is evident. **c** Subtype II is significantly enriched for large-cell anaplastic histology. **d** No difference in metastatic disease (i.e., M0 vs M1+ disease) between subgroups. *p* values shown are derived from Chi squared tests of enrichment performed across all subtypes

The clinico-pathological and molecular correlates of the concordant Grp3 and Grp4 subtypes are shown in Fig. 5. The multi-modal age-of-incidence profiles of Grp3 and Grp4 are now resolved by their classification into eight distinct subtypes (Fig. 5a). Subtype VII is bimodal and is resolved into two distinct age distributions by its distinction into VII-A/VII-B (supplementary Fig. 7). Subtype V also has a bimodal age distribution which cannot currently be resolved by further subdivision of this subtype. Subtype IV is predominantly infant, whereas subtype VIII peaks at 10 years of age. We observed no differences in biological sex between subtypes (Fig. 5b); however, LCA histopathological variants were significantly enriched in subtype II [24/54 (44%), *p* < 0.0001]. Interestingly, we also observed infrequent tumors with DN/MBEN histology [45/631 (7%) with available data] across all variants except subtype III. This is unlikely to be due entirely to miscalling by local pathologists—of the 9 cases arising from the Schwalbe et al. cohort with available histology, 8 of them had been centrally reviewed, thus confirming that DN/MBEN histology does not automatically confer an SHH molecular subgroup (Fig. 5c). Subtype V was most commonly metastatic [34/54 (61%)], although this was not significantly enriched compared to other subtypes (Fig. 5d).

### Subtype-specific survival

**Fig. 6:**
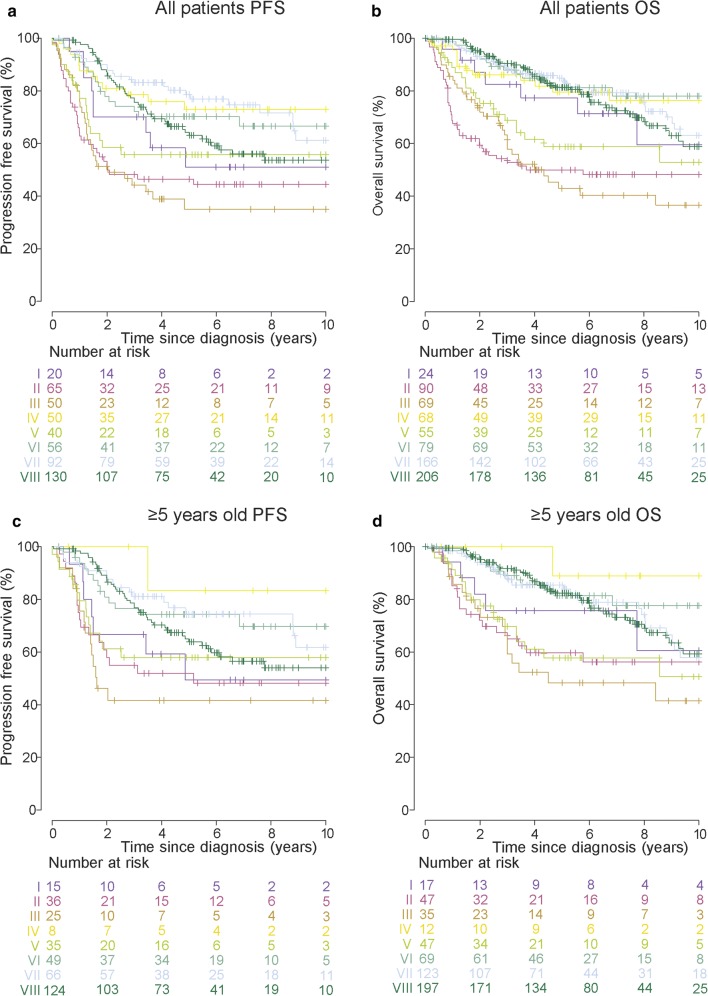
Grp3/4 subtypes have distinct survival outcomes. Kaplan–Meier plots are shown for PFS/OS for all samples with available survival data (**a**, **b**), and to avoid confounding by patients treated with infant protocols, a filtered cohort comprised of patients aged ≥ 5 years at diagnosis (**c**, **d**). The at-risk table below the Kaplan–Meier plots (I–VIII) shows the number of patients at risk at specific times after diagnosis (0–10 years in 2 year intervals)

PFS (*n* = 550) and OS (*n* = 837) were analyzed from all assessable patients (Fig. 6); PFS was unavailable for the Cavalli et al. data set. The eight Grp3 and Grp4 molecular subtypes can be classified into three distinct risk groups based on their overall survival. The first, a very high-risk group (5-year OS 50%, 95% CI 43–58), contains subtypes II, III, and V. Groups II and III, enriched for *MYC* amplification, showed equivalent poor survival [5-year OS 50% (95% CI 40–62) and 43% (95% CI 32–59), respectively]. Subtype V, enriched for *MYC*/*MYCN* amplification, also showed a poor survival (5-year OS 59%, 95% CI 46–75).

The second group is associated with late/relapse death and comprises solely of subtype VIII, this feature is unique to this subtype within MB and consistent with the previous reports [26, 32]; of 206 assessable subtype VIII patients, 17/49 (35%) deaths occurred ≥5 years after diagnosis.

The final group is standard risk (5-year OS 82%, 95% CI 78–87) and comprises subtypes I (5-year OS 77%, 95% CI 62–97), IV (80%, 95% CI 70–91), VI (81%, 95% CI 72–91), and VII (85%, 95% CI 79–91). Considering PFS, subtype I had a high-risk 5-year PFS (51%, 95% CI 32–81); however, this was the smallest group (*n* = 20), and this difference may be due to sampling; all the other subtypes had similar PFS and OS patterns.

Significant cohort-specific survival differences were observed. For OS, patients from the Schwalbe et al. cohort had significantly lower survival (5-year OS 64%, 95% CI 58–71) than the Cavalli et al. (5-year OS 74%, 95% CI 68–80) and extended Northcott et al. (5-year OS 79%, 95% CI 74–84) collections (supplementary Fig. 10). Stratification by subtype revealed that this difference was subtype-specific and that only in subtype VIII were poorer outcomes significantly associated with membership of the Schwalbe et al. cohort (HR 3.87 relative to Northcott et al. cohort, 95% CI 1.85–8.10, *p* = 0.00033) (data not shown). To avoid confounding by patients treated with infant protocols, we excluded patients aged ≤ 5 years and observed similar survival patterns to those described above.

### Refinement of Grp3/Grp4 molecular classification

**Fig. 7:**
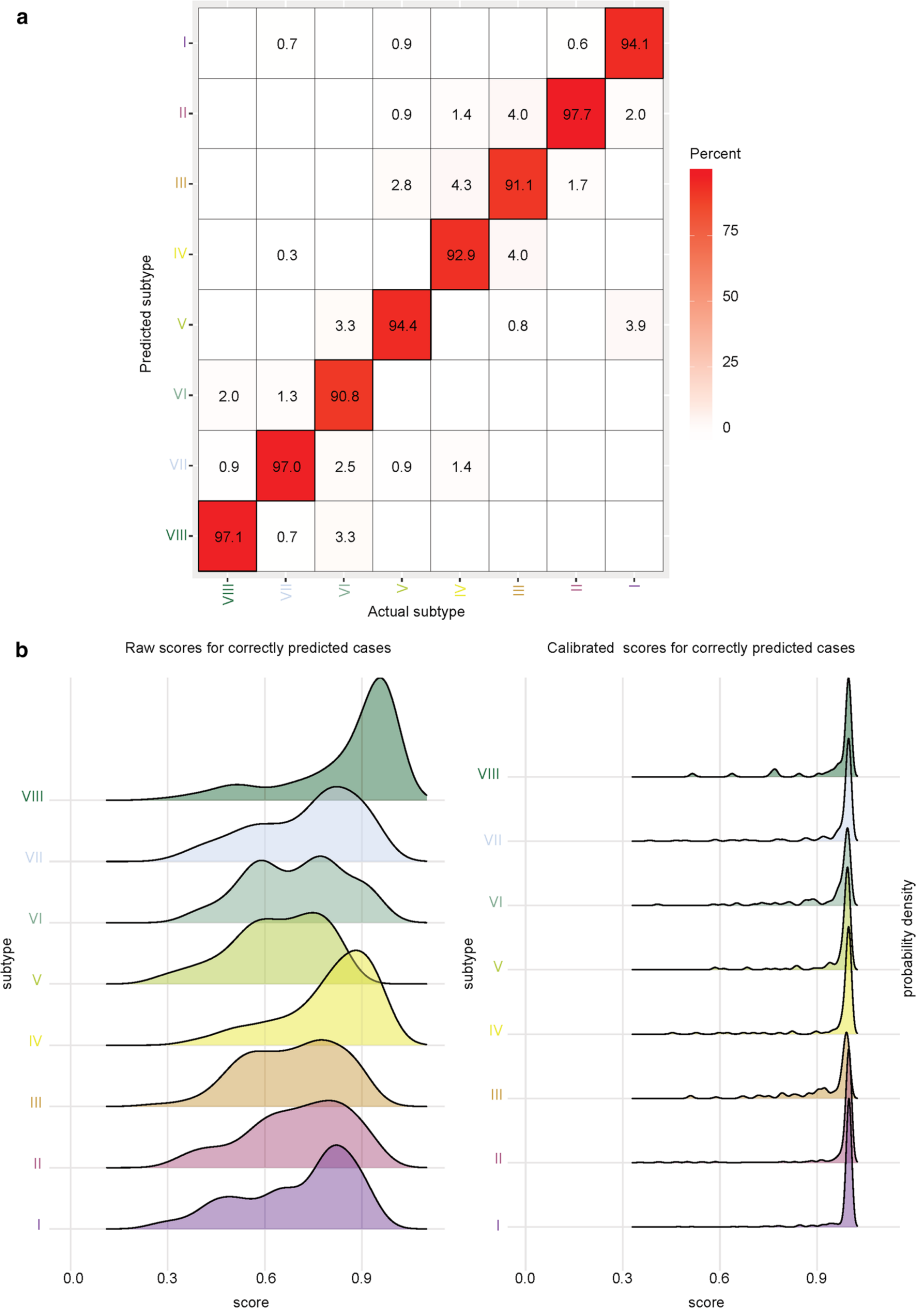
Molecular classifier for Grp3/4 subtype identification. **a** Confusion matrix shows relationship between predicted and actual subtype calls for the Grp3/4 cohort. The minimum reproducibility across subtypes was > 90%. **b** Density plots show raw and calibrated scores from classifier (refer to supplementary methods for detailed information). After calibration, the prediction accuracy increased, with an overall error rate of 4.6%

To prospectively assign cases to Grp3/Grp4 concordant subtypes, we developed an extension of the Heidelberg brain tumor classifier [2]. This classifier was evaluated by threefold nested cross validation, and performance measures revealed a misclassification error of 4.6%, a corresponding Area under the curve (AUC) of 0.9969 and Brier Score of 0.069 (for more details, refer to the supplementary methods). More than 85% of correctly classified samples (1166/1370) achieved a high calibrated score > 90% (class probability estimates), indicating that this might represent a rational cutoff to define high confidence predictions in diagnostic applications (Fig. 7). Subtypes III (91.1% concordant) and VI (90.8% concordant) were the most difficult to resolve. This classifier is publicly available at [https://www.molecularneuropathology.org/mnp/classifier/7].

## Discussion

Following the recommendations of the 2012 consensus paper [19, 29], we worked together in a multi-center, collaborative analysis that should become standard practice in the future. We assembled the largest cohort (*n* = 1501) of Grp3 and Grp4 medulloblastoma analyzed to date. Instead of advocating one single approach or method, we applied multiple class-definition approaches and resampling measures to assess subtype stability, robustness, and reproducibility. We also gave equal weight to each analytical technique to ensure that the identified subtypes were a true accord (Fig. 1).

The lowest complexity solutions continued to identify Grp3 and Grp4, as described in the original consensus. However, objective subclassification most strongly supported further robust and reproducible subtypes within them. We identified these subtypes by mapping subtype assignments across studies (Fig. 2). Five out of eight subtypes were concordant between ≥ 2/3 studies. The other three subtypes were initially discrepant and additionally required the assessment of associated clinico-biological features to specify optimal subtype definitions. We identified eight subtypes [I–VIII, with additional evidence that VII is comprised of two variants, VII-A and VII-B (supplementary Fig. 7)]. No evidence was found to support additional subtypes beyond eight, using a cohort totaling > 1500 tumors and currently available genomic readouts.

Subtypes I, V, and VII were mixed Grp3 and Grp4 in composition, indicating shared Grp3 and Grp4 biology. Of note, absolute separation between Grp3 and Grp4 was observed when using SNF on paired methylation and transcriptomic data, but was not observed when applying SNF to DNA-methylation data alone. Data and platform limitations precluded equivalently comprehensive assessment of the contribution of transcriptomics to the separation of Grp3 and Grp4. Transcriptomic and DNA methylomic patterns may interact to further define subclasses and investigations to account for these differences are now required.

**Fig. 8:**
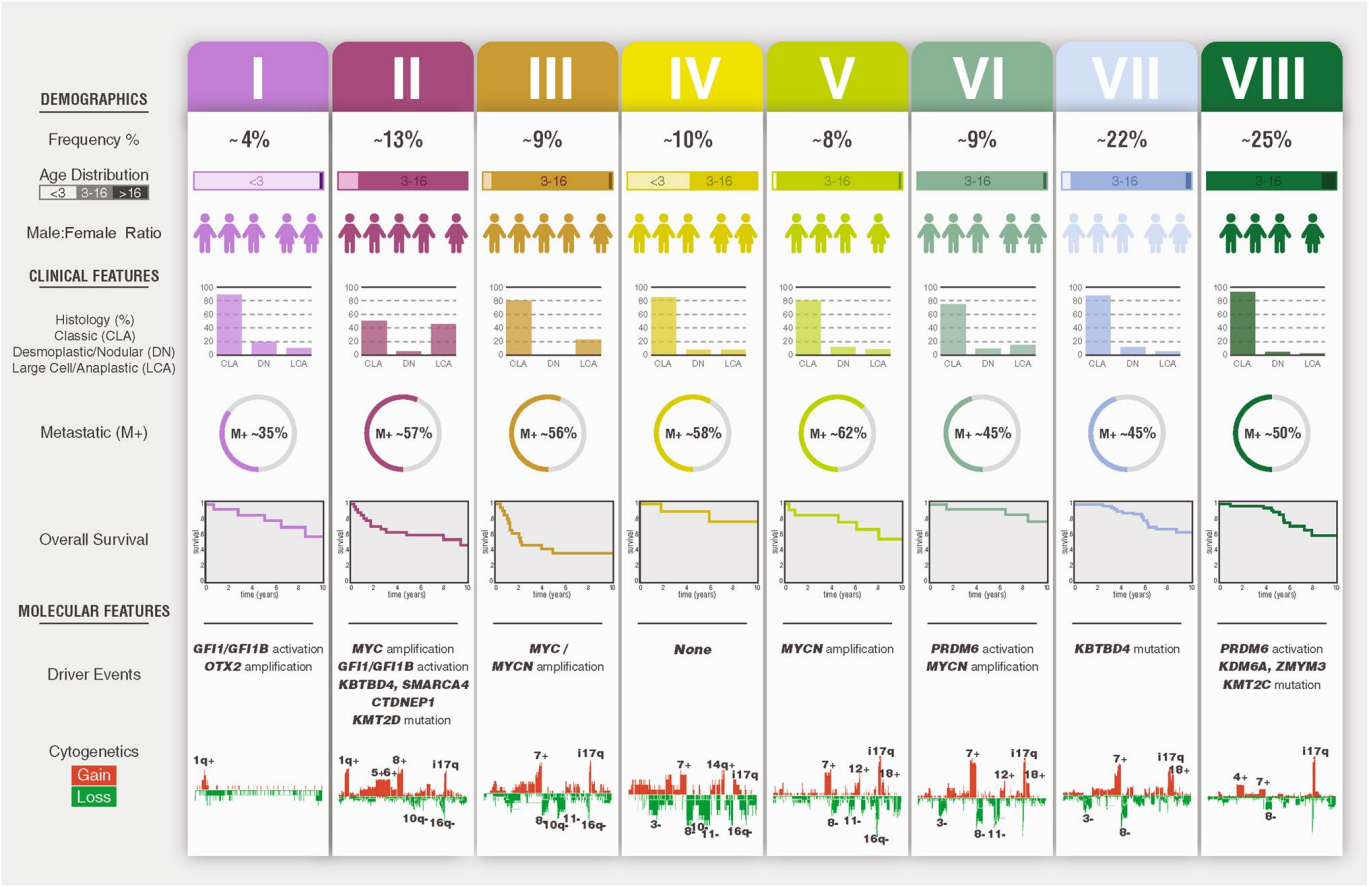
Summary of molecular subtypes of Grp3/4 medulloblastoma. The major demographic, clinico-pathological, and molecular features of the concordant subtypes are summarized. Mutation data were derived solely from Northcott et al. [18]. For histology, *CLAS* classic, *DN* desmoplastic nodular, *LCA* large-cell anaplastic. M+ (%) = metastatic (i.e., M1+) frequency. Overall survival shows subtype-specific survival in years. Cytogenetic gains are shown in red, losses in green

Overall, the distinction into eight subtypes (Fig. 8) was further supported by their significant cytogenetic differences–each subtype is associated with specific cytogenetic signatures. However, cytogenetic signatures in isolation are insufficient to identify the eight subtypes, and the subtype-specific methylation changes we observed are not enriched in chromosomes with frequent subtype-specific chromosomal copy-number aberrations (supplementary Fig. 11).

The CNV correlates in most subtypes were as expected [18], e.g., enrichment for *MYC* amplification in subtype II and *MYC*/*MYCN* amplification in III/V, respectively. Unlike *CTNNB1* mutation in MB_WNT_ tumors, which is near universal within that subgroup, the alteration of no one gene, or set of genes, is able to fully describe a single methylation-dependent subtype. Rather, specific methylation subtypes are associated with significant enrichment for particular mutational/amplification events.

The subtypes (Fig. 8) show distinct ages of incidence. Although most are unimodal, subtypes V and VII are bimodal. Subtype VII can be resolved into VII-A and VII-B (supplementary Fig. 7), each with distinct ages of incidence. The age distribution for V may, therefore, indicate overlapping distributions of two further, as yet undefined, distinct groups. The survival analyses demonstrated that there is substantial divergence in outcomes between subtypes and that the association of Grp3 with a universally poor prognosis reported in the original consensus study in 2012 requires further clinical definition and revision, since Grp3 encompasses the *MYC*-enriched subtypes II and III, with a poor survival in this cohort (5-year OS 49 and 41%, respectively), in addition to subtype IV, which is standard risk (5-year OS 80%), consistent with Schwalbe et al. [26].

In future studies, it will be essential to assess the relative contributions and interactions between the original consensus subgroups, their novel subtypes, clinical factors, and other concerted molecular features (e.g., whole chromosome and focal cytogenetic aberrations), including established disease risk factors, in driving disease development and clinical behavior. For example, recently described non-random whole-chromosome aberration signatures encompassing loss of chromosomes 8 and 11 and gain of chromosome 7, which confer a favorable prognosis in standard-risk Grp3/4 medulloblastomas [11], show clear associations with subtypes IV, VI, and VII and implicate common biology in their development.

The survival analysis also identified the phenomenon of subtype VIII-associated late relapse in medulloblastoma. The reasons for this need to be urgently explored; careful monitoring of subtype VIII survivors should be considered to identify relapse as quickly as possible and distinguish this from the occurrence of secondary malignancies. Since this is the largest subtype, the acquisition of large cohorts suitable for the investigation of this question is feasible in the short term.

Nevertheless, these survival investigations are not without their limitations. These data are collected from diverse treatment centers, and patients have received heterogeneous therapy which may confound the relationships described here, although based on our current data, we have the first indications that these subtypes show differential survival.

The new Grp3 and Grp4 classifier that we developed indicates that these subtypes are readily distinguishable and demonstrates how these subtypes could be identified and their clinical utility assessed in future studies. These could include the prospective investigation of the clinical impact of current and newly described disease risk biomarkers in this context, identification of their underpinning biology and the potential for novel therapeutic interventions. For now, recognition of the low-risk Grp3 subtype (IV), and late relapses in subtype VIII patients represents strong candidates for future clinical investigation and advancement.

Novel subtypes resolved in our analysis are primarily based on DNA-methylation profiles with support from transcriptomic data sets. It has been suggested that the differing methylation signatures observed in medulloblastoma are a reflection of the developmental state of the cell of origin at time of tumorigenesis [31]. It would be interesting to investigate whether the subtypes with contributions from Grp3 and Grp4 (i.e., I, V, and VII) share a common cell of origin, and, if so, how this might relate to any transcriptional differences between Grp3 and Grp4 variants of the same subtype. There is also potential to integrate our current results with new approaches and explore further resolution of disease subclassification (e.g., the proteomic and transcriptomic signatures of these novel subtypes and their constitution at the single-cell level); the first descriptions of the medulloblastoma proteome [1, 10] describe additional heterogeneity within Grp3 and Grp4 and seem to identify subtype II as a distinct Grp3 high-risk subtype.

This study lays the groundwork to robustly assess additional heterogeneity within Grp3 and Grp4 medulloblastoma, to define its biological and clinical relevance, and to exploit these in improved therapeutic approaches.

## Electronic supplementary material

Below is the link to the electronic supplementary material.

Supplementary material 1 (PDF 3954 kb)

## Data Availability

The methylation data analyzed for this paper is available from GEO (https://www.ncbi.nlm.nih.gov/geo/) with the identifier GSE130051.
