# Supplementary material for: Second-generation molecular subgrouping of medulloblastoma: an international meta-analysis of Group 3 and Group 4 subtypes
Source: Acta Neuropathol. 2019 May 10;138(2):309–26. doi: 10.1007/s00401-019-02020-0 (PMC6660496; doi:10.1007/s00401-019-02020-0)
Supplement: Supplementary file 1 — Supplementary material 1 (PDF 3954 kb) [file 401_2019_2020_MOESM1_ESM.pdf]

Second-generation molecular subgrouping of medulloblastoma: an international meta-analysis of Group 3 and Group 4 subtypes

T Sharma, E C Schwalbe et al.

Appendix

Supplementary Methods

We undertook a retrospective, integrative analysis of Grp3/4 substructure from three published component studies [1-3], whose respective major findings are summarized in figure 1. Quality control checks revealed no biases attributable to sample type (*i.e.* fresh frozen vs formalin-fixed, paraffin embedded derivatives), array type (450k vs 850k), or source study/institution (supplementary Figure 3). 69 previously published samples were removed due to low methylation classifier prediction scores [4] and 80 samples were additionally removed due to shared genotypes (i.e. duplicate samples/patients). 28 samples were removed due to a 'non-MB' prediction by the methylation classifier (supplementary Tables 1 and 2; supplementary Figure 2. The cohorts and their demographics are summarized in Table 1.

MB classifier

Before training the classifier, a correction for the type of material tissue (FFPE or frozen) and array (450k or EPIC) was performed by fitting a linear model to the log2-transformed intensity values (removeBatchEffect function, limma package v.3.36 [5] including the material tissue and array type as factor variables. The methylated and unmethylated signals were corrected individually. Estimated batch effects were also used to adjust diagnostic samples or test samples within the cross-validation. Beta values were calculated from the retransformed intensities using an offset of 100 (as recommended by Illumina).

To train the RF classifier, the randomForest R package [6] was used. For feature selection, the 50,000 CpG probes with highest SD were selected. In a second step, an RF was trained to calculate a RF permutation-based variable importance measure for the selected CpG probes. Finally, the 10,000 CpG probes with highest variable importance were used to train the final RF model. For both RF models 5,000 trees were fitted and imbalanced class sizes were accounted for by down-sampling to the minority class; this ensures an identical number of samples per class and tree [7]. The performance of the RF model was validated by a threefold cross-validation, validating in each fold all steps of the classifier development, starting with the batch adjustment.

The classification scores generated by the RF model (that is, the proportion of trees voting for a class) perform already well when used to predict the correct Grp3/4 subtype, but they do not reflect well calibrated class probabilities that may guide clinical decision making [6]. Furthermore, the distribution of the RF scores may vary between classes, which make inter-class comparisons difficult. To obtain scores that are comparable between classes and that are improved estimates of the certainty of individual predictions, we performed a classification score recalibration by mapping the original scores to more accurate class probabilities[8]. To find such a mapping, an L2-penalized, multinomial, logistic regression model was fitted, which takes the methylation class as response variable and the RF scores as explanatory variables. The R package glmnet [9] was used to fit this model. In addition, the model was fitted by incorporating a small ridge-penalty (L2) on the likelihood to prevent overfitting, as well as to stabilize estimation in situations in which classes are perfectly separable. The amount of this regularization, that is, the penalization parameter, is determined by running a tenfold cross-validation and choosing penalization parameter that minimizes the mean squared error (MSE). Independent RF scores are needed to fit this model, that is, the scores need to be generated by a RF classifier that was not trained using the same samples, otherwise the RF scores will be systematically biased and not comparable to scores of unseen cases. As such, RF scores generated by the threefold cross-validation are used. To validate the class predictions generated by using the recalibrated scores of the calibration model, an additional, nested threefold cross-validation loop is incorporated into the main threefold cross-validation. Within each

cross-validation run this nested threefold cross-validation is applied to generate independent RF scores, which are then used to train a calibration model. The predicted RF scores resulting from predicting the one-third test data of the outer cross-validation loop are then recalibrated by applying the calibration model that was fitted on the RF scores generated on the other two-third of the data by the nested cross-validation. Performances of the resulting classifier predictions and scores generated by the cross-validation were assessed by the misclassification error, multiclass area under receiver operating characteristic (ROC) curve (AUC) and the multiclass Brier score. The misclassification error measures the frequency of falsely assigned class labels when using the maximum of the RF scores or re-calibrated scores as a cutoff to determine the predicted class, that is, the majority vote. To measure the AUC for our multiclass RF the generalization of the AUC for multiclass classification problems[10] was used. To measure how well the resulting RF scores and recalibrated scores perform when used as class probabilities, the multiclass Brier score was used [11,12].

References:

1 Cavalli FMG, Remke M, Rampasek L, Peacock J, Shih DJH, Luu B et al. (2017) Intertumoral Heterogeneity within Medulloblastoma Subgroups. Cancer Cell 31: 737-754 e736

2 Northcott PA, Buchhalter I, Morrissy AS, Hovestadt V, Weischenfeldt J, Ehrenberger T et al. (2017) The whole-genome landscape of medulloblastoma subtypes. Nature 547: 311-317

3 Schwalbe EC, Lindsey JC, Nakjang S, Crosier S, Smith AJ, Hicks D et al. (2017) Novel molecular subgroups for clinical classification and outcome prediction in childhood medulloblastoma: a cohort study. Lancet Oncol 18: 958-971

4 Capper D, Jones DTW, Sill M, Hovestadt V, Schrimpf D, Sturm D et al. (2018) DNA methylation-based classification of central nervous system tumours. Nature 555: 469-474

5 Ritchie ME, Phipson B, Wu D, Hu Y, Law CW, Shi W, Smyth GK (2015) limma powers differential expression analyses for RNA-sequencing and microarray studies. Nucleic Acids Res 43: e47

6 Simon R (2014) Class probability estimation for medical studies. Biom J 56: 597-600

7 Chen CL, A.; Breiman, L. (2004) Report 666: Using Random Forest to Learn Imbalanced Data. City

8 Bostrom H (2008) Calibrating Random Forests. City

9 Friedman J, Hastie T, Tibshirani R (2010) Regularization Paths for Generalized Linear Models via Coordinate Descent. J Stat Softw 33: 1-22

10 Hand DJ, Till RJ (2001) A simple generalisation of the area under the ROC curve for multiple class classification problems. Machine Learning 45: 171-186

11 Brier GW (1950) Verification of Forecasts Expressed in Terms of Probability. Monthly Weather Review 78: 1-3

12 Kim KI, Simon R (2014) Overfitting, generalization, and MSE in class probability estimation with high-dimensional data. Biom J 56: 256-269

Idat	Study_ID	Derivative Material	Array	Study	Prediction	Score
200788260028_R06C01	93622	FFPE	EPIC	Northcott	MB, G4	0.179
200397860036_R06C01	P036_90802_diag	Fresh-frozen	450k	Northcott	MB, G3	0.085
200784530098_R06C01	P036_93530_diag	FFPE	EPIC	Northcott	MB, G3	0.449
7970376085_R02C01	TO_MB10	Fresh-frozen	450k	Northcott	MB, G3	0.829
7970376048_R01C01	TO_MB1	Fresh-frozen	450k	Northcott	MB, G3	0.829
7810920080_R04C01	TO_MB33	Fresh-frozen	450k	Northcott	MB, G3	0.368
7970376149_R04C01	TO_MB5	Fresh-frozen	450k	Northcott	MB, G3	0.705
7973219019_R04C02	MB-0580	Fresh-frozen	450k	Northcott	MB, G4	0.787
8942326039_R01C02	MB-2631	Fresh-frozen	450k	Northcott	MB, G3	0.633
7970376151_R01C01	MB-MT1179	Fresh-frozen	450k	Northcott	MB, G3	0.877
7796806110_R01C01	MB-MT314	Fresh-frozen	450k	Northcott	MB, G3	0.695
7810920068_R02C01	ID_7810920068_R02C01	Fresh-frozen	450k	Cavalli	MB, G4	0.688
7810920071_R01C01	ID_7810920071_R01C01	Fresh-frozen	450k	Cavalli	MB, G4	0.898
7810920078_R05C02	ID_7810920078_R05C02	Fresh-frozen	450k	Cavalli	MB, G4	0.661
7810920079_R05C01	ID_7810920079_R05C01	Fresh-frozen	450k	Cavalli	MB, G4	0.818
7970368003_R01C01	ID_7970368003_R01C01	Fresh-frozen	450k	Cavalli	MB, G3	0.818
7970368007_R02C01	ID_7970368007_R02C01	Fresh-frozen	450k	Cavalli	MB, G4	0.877
7970368045_R06C02	ID_7970368045_R06C02	Fresh-frozen	450k	Cavalli	MB, G4	0.897
7970368069_R04C01	ID_7970368069_R04C01	Fresh-frozen	450k	Cavalli	MB, G3	0.769
7970368130_R04C02	ID_7970368130_R04C02	Fresh-frozen	450k	Cavalli	MB, G4	0.776
7970376002_R04C01	ID_7970376002_R04C01	Fresh-frozen	450k	Cavalli	MB, G4	0.871
7970376148_R02C01	ID_7970376148_R02C01	Fresh-frozen	450k	Cavalli	MB, G4	0.737
7973201069_R04C02	ID_7973201069_R04C02	Fresh-frozen	450k	Cavalli	MB, G4	0.670
7973201149_R04C01	ID_7973201149_R04C01	Fresh-frozen	450k	Cavalli	MB, G3	0.440
7973201166_R02C01	ID_7973201166_R02C01	Fresh-frozen	450k	Cavalli	MB, G3	0.383
7973219003_R01C01	ID_7973219003_R01C01	Fresh-frozen	450k	Cavalli	MB, G3	0.493
7973219008_R04C01	ID_7973219008_R04C01	Fresh-frozen	450k	Cavalli	MB, G4	0.899
7973219018_R01C01	ID_7973219018_R01C01	Fresh-frozen	450k	Cavalli	MB, G3	0.860
7973219040_R04C02	ID_7973219040_R04C02	Fresh-frozen	450k	Cavalli	MB, G4	0.875
8942326054_R03C02	ID_8942326054_R03C02	Fresh-frozen	450k	Cavalli	MB, G3	0.481
9326189004_R03C02	ID_9326189004_R03C02	Fresh-frozen	450k	Cavalli	MB, G4	0.758
9326189013newLazer_R01C01	ID_9326189013newLazer_R01C01	Fresh-frozen	450k	Cavalli	MB, G4	0.882
9326189013newLazer_R03C01	ID_9326189013newLazer_R03C01	Fresh-frozen	450k	Cavalli	MB, G4	0.529
9326189023_R06C01	ID_9326189023_R06C01	Fresh-frozen	450k	Cavalli	MB, G4	0.798
9326189024newLazer_R03C01	ID_9326189024newLazer_R03C01	Fresh-frozen	450k	Cavalli	MB, G4	0.746
9326189024newLazer_R04C02	ID_9326189024newLazer_R04C02	Fresh-frozen	450k	Cavalli	MB, G3	0.491
9326189025newLazer_R04C02	ID_9326189025newLazer_R04C02	Fresh-frozen	450k	Cavalli	MB, G3	0.854
9341679041_R06C01	ID_9341679041_R06C01	Fresh-frozen	450k	Cavalli	MB, G4	0.610
9344737005_R01C01	ID_9344737005_R01C01	Fresh-frozen	450k	Cavalli	MB, G3	0.656
9344737005_R04C02	ID_9344737005_R04C02	Fresh-frozen	450k	Cavalli	MB, G3	0.583
9344737044_R06C01	ID_9344737044_R06C01	Fresh-frozen	450k	Cavalli	MB, G4	0.878
9344737073_R01C02	ID_9344737073_R01C02	Fresh-frozen	450k	Cavalli	MB, G3	0.332

9421912024_R01C01	NMB119	Fresh-frozen	450k	Schwalbe	MB, G4	0.731
9421912022_R01C02	NMB269	Fresh-frozen	450k	Schwalbe	MB, G3	0.721
9647455134_R01C01	NMB325	FFPE	450k	Schwalbe	MB, G4	0.488
7970368088_R05C01	NMB440	Fresh-frozen	450k	Schwalbe	MB, G3	0.721
7973201123_R06C02	NMB445	Fresh-frozen	450k	Schwalbe	MB, G3	0.533
9647455136_R04C01	NMB476	Fresh-frozen	450k	Schwalbe	MB, G3	0.733
9421912041_R01C01	NMB49	Fresh-frozen	450k	Schwalbe	MB, G4	0.889
6229017107_R01C02	NMB583	Fresh-frozen	450k	Schwalbe	MB, G4	0.519
7973201167_R01C02	NMB717	Fresh-frozen	450k	Schwalbe	MB, G4	0.522
9403904011_R01C02	NMB795	Fresh-frozen	450k	Schwalbe	MB, G3	0.697
7970368034_R06C02	PNET30032	FFPE	450k	Schwalbe	MB, G4	0.617
9647455150_R05C01	PNET350120	FFPE	450k	Schwalbe	MB, G3	0.239
9647455134_R02C02	PNET350241	FFPE	450k	Schwalbe	MB, G4	0.801
8622007029_R01C02	5M3	Fresh-frozen	450k	Northcott	MB, G4	0.686
9374341006_R05C02	ICGC_MB223	Fresh-frozen	450k	Northcott	MB, G3	0.884
9406921070_R01C02	P036_64906_diag	FFPE	450k	Northcott	MB, G4	0.180
9553932002_R05C01	AK_2652_12	FFPE	450k	Northcott	MB, G3	0.791
9610361029_R06C02	AK_4480_08	FFPE	450k	Northcott	MB, G3	0.809
9610361022_R06C02	AK_4981_10	FFPE	450k	Northcott	MB, G3	0.516
9761749088_R02C02	ICGC_MB289	Fresh-frozen	450k	Northcott	MB, G3	0.861
9968646163_R05C02	P036_69190_ref	FFPE	450k	Northcott	MB, G3	0.462
9969477090_R04C01	ICGC_MB291	Fresh-frozen	450k	Northcott	MB, G3	0.787
3999543042_R02C02	P036_75374_ref	FFPE	450k	Northcott	MB, G4	0.384
3998920116_R05C02	T51_MB	Fresh-frozen	450k	Northcott	MB, G3	0.804
9741950157_R05C02	SJMB03-UPN10669654	FFPE	450k	SJMB03	MB, G3	0.707
9741950172_R01C01	SJMB03-UPN13648866	FFPE	450k	SJMB03	MB, G3	0.849
9934987066_R04C01	SJMB03-UPN18600500	FFPE	450k	SJMB03	MB, G4	0.211

Supplementary table 1. List of samples excluded from study due to low classifier prediction score using Heidelberg molecular neuropathology brain tumour classifier. For each sample, its IDAT identifier, study ID, study, provisional classification and prediction score are given.

Idat	Study ID	Derivative Material	Array	Study	Prediction	Score
200498360016_R03C01	91426	FFPE	EPIC	Northcott	CONTR, CEBM	0.497
200790260040_R07C01	P036_93488_diag	Fresh-frozen	EPIC	Northcott	GBM, RTK I	0.318
7970368034_R04C01	NMB625	FFPE	450k	Schwalbe	ATRT, SHH	0.999
9647455136_R02C01	NMB128	Fresh-frozen	450k	Schwalbe	CONTR, CEBM	0.999
6222421011_R05C02	NMB168	Fresh-frozen	450k	Schwalbe	CONTR, CEBM	0.998
7970368035_R02C02	NMB437	Fresh-frozen	450k	Schwalbe	GBM, MYCN	0.416
7970368032_R04C01	NMB662	FFPE	450k	Schwalbe	ETMR	0.998
7970368052_R06C02	NMB724	Fresh-frozen	450k	Schwalbe	ETMR	0.996
9422491011_R03C02	NMB869	Fresh-frozen	450k	Schwalbe	GBM, MID	0.511
9647450105_R06C02	PNET30035	FFPE	450k	Schwalbe	EPN, PF A	0.942
9403904116_R02C02	NMB63	Fresh-frozen	450k	Schwalbe	CONTR, CEBM	0.957
7973201123_R01C02	NMB441	Fresh-frozen	450k	Schwalbe	PLEX, PED B	0.758
9647455137_R03C02	NMB823	FFPE	450k	Schwalbe	GBM, MID	0.822
7970368138_R02C02	NMB345	FFPE	450k	Schwalbe	ATRT, SHH	0.383
9403904132_R05C02	NMB400	Fresh-frozen	450k	Schwalbe	CONTR, CEBM	0.945
7973201008_R03C02	NMB759	Fresh-frozen	450k	Schwalbe	ETMR	0.787
9647455134_R06C01	PNET350049	FFPE	450k	Schwalbe	PLEX, PED B	0.487
9647455169_R03C01	NMB485	Fresh-frozen	450k	Schwalbe	PLEX, PED B	0.192
7970368032_R03C02	NMB617	FFPE	450k	Schwalbe	O IDH	0.319
9305651213newLazer_R06C01	MB-0174	Fresh-frozen	450k	Northcott	CONTR, HEMI	0.999
7970376150_R06C01	MB-1351	Fresh-frozen	450k	Northcott	RETB	0.589
9326189011newLazer_R04C02	MB-2897	Fresh-frozen	450k	Northcott	SUBEPN, SPINE	0.340
7810920078_R06C01	ID_7810920078_R06C01	Fresh-frozen	450k	Cavalli	DMG, K27	0.420
7970368124_R03C01	ID_7970368124_R03C01	Fresh-frozen	450k	Cavalli	CONTR, CEBM	0.665
8959312036_R02C02	ID_8959312036_R02C02	Fresh-frozen	450k	Cavalli	CONTR, CEBM	0.983
9326189009newLazer_R06C01	ID_9326189009newLazer_R06C01	Fresh-frozen	450k	Cavalli	CONTR, CEBM	0.298
9344737044_R06C02	ID_9344737044_R06C02	Fresh-frozen	450k	Cavalli	CONTR, CEBM	0.437
7973201069_R01C02	ID_7973201069_R01C02	Fresh-frozen	450k	Cavalli	PLEX, PED B	0.301

Supplementary table 2. List of samples excluded from study due to non-medulloblastoma prediction using Heidelberg molecular neuropathology brain tumor classifier. For each sample, its IDAT identifier, study ID, study, provisional classification and prediction score are given.

Supplementary Table 3. Inter-technique mapping between subtype assignments.

tSNE	NMF	SNF	Agreement
I	Grp3.HR.2	-	2/3
II	Grp3.HR.1	5	3/3
III	Grp3.LR.2/Grp3.LR.1	2	2/3
IV	Grp3.LR.2/Grp3.LR.1	6	2/3
V	Grp4.LR2.2	-	2/3
VI	Grp4.LR2.1	-	2/3
VII	Grp4.LR1.1<br>Grp4.LR1.2	7<br>4	2/3<br>2/3
VIII	Grp4.HR	-	2/3

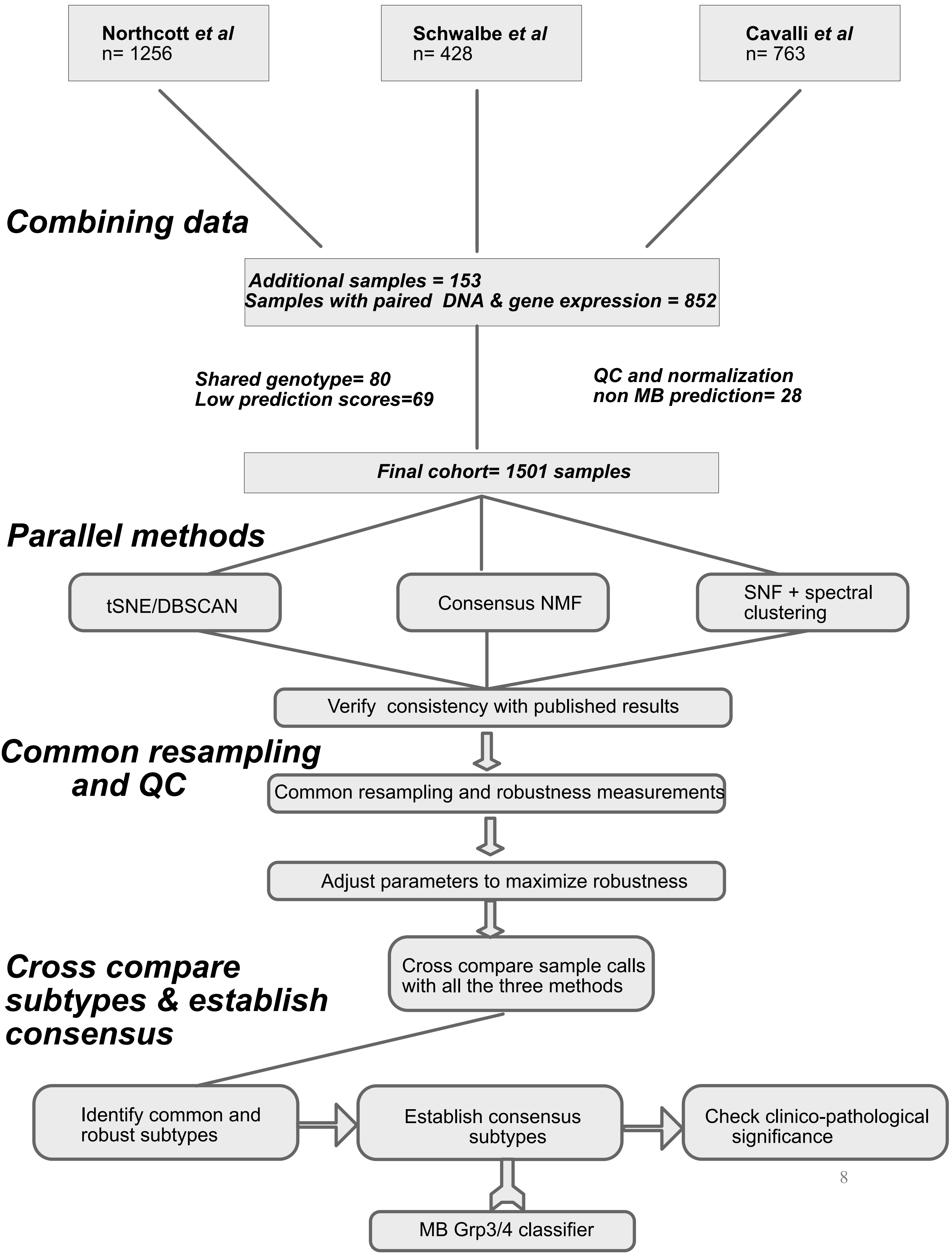
Northcott et al
n= 1256
Schwalbe et al
n= 428
Cavalli et al
n= 763
Combining data
Additional samples = 153
Samples with paired DNA & gene expression = 852
Shared genotype= 80
Low prediction scores=69
QC and normalization
non MB prediction= 28
Final cohort= 1501 samples
Parallel methods
tSNE/DBSCAN
Consensus NMF
SNF + spectral clustering
Verify consistency with published results
Common resampling and QC
Common resampling and robustness measurements
Adjust parameters to maximize robustness
Cross compare subtypes & establish consensus
Cross compare sample calls with all the three methods
Identify common and robust subtypes
Establish consensus subtypes
Check clinico-pathological significance
MB Grp3/4 classifier

Supplementary Figure 1. Flowchart shows sequence of analysis to define and characterise consensus subgroups of Group 3/4 medulloblastoma

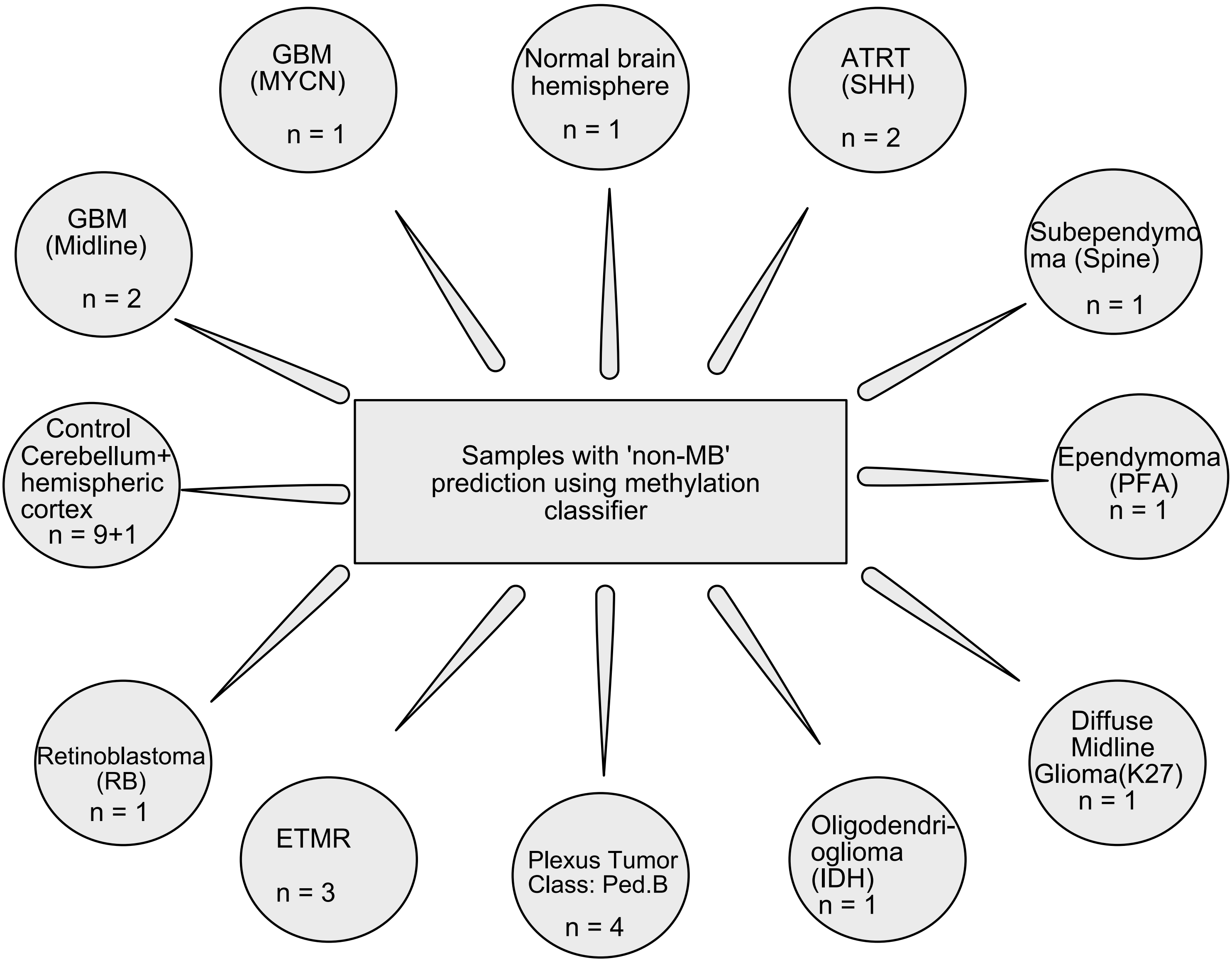

Supplementary Figure 2. Classification of study-specific samples designated as non-MB by methylation classifier. The detailed description of each of the classes can be found at : https://www.molecularneuropathology.org/mnp/classifier/2

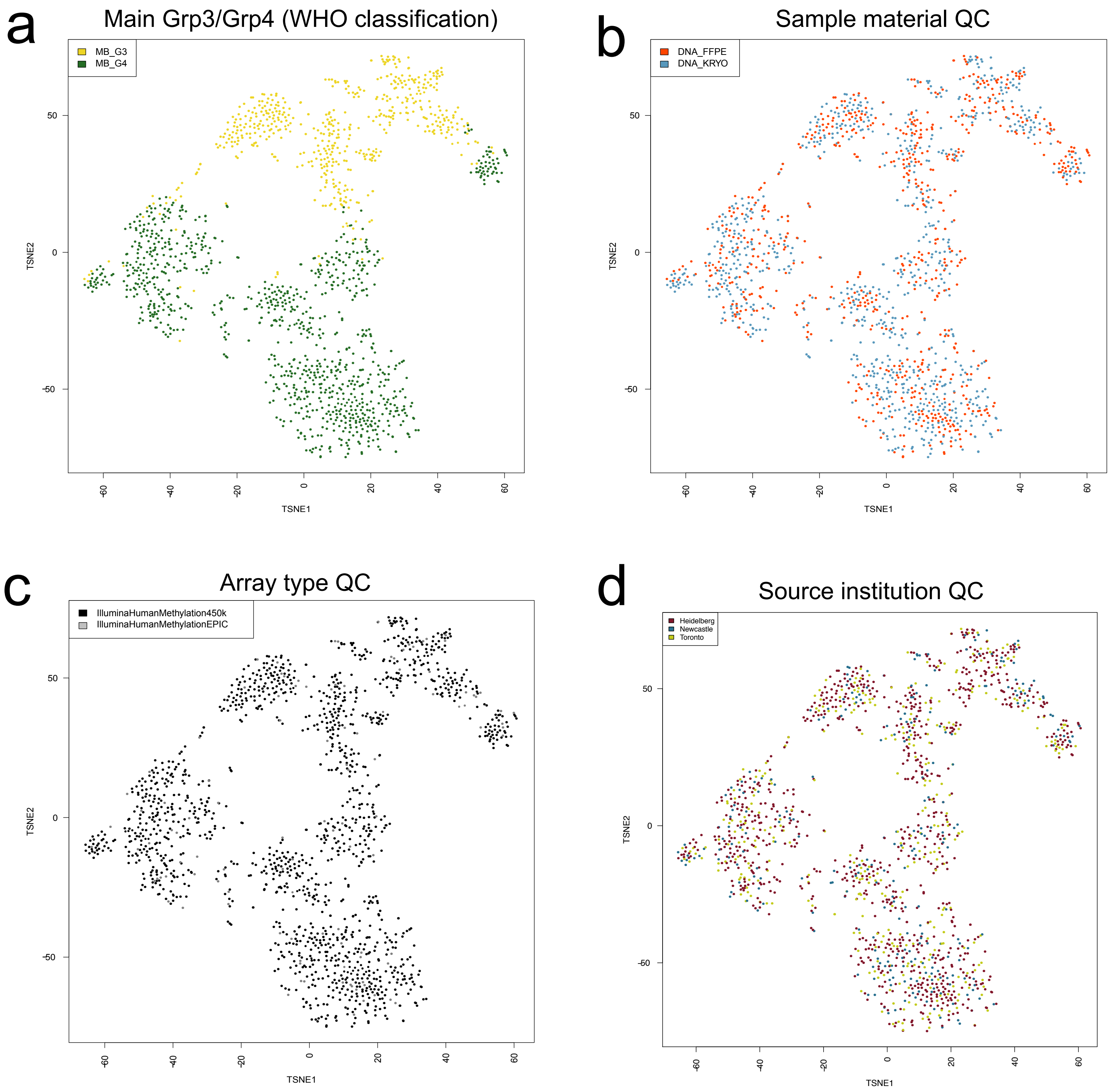

Supplementary Figure 3. Quality control investigations reveal no bias. a. t-SNE plot of entire Grp3/Grp4 cohort coloured by Grp3/4 classification (Grp3, yellow; Grp4, green) by Heidelberg MNP2.0 classifier. **b.** t-SNE plot of entire Grp3/4 cohort coloured by derivative type (fresh-frozen (KRYO), blue; formalin-fixed, paraffin embedded (FFPE), red). **c.** t-SNE plot of entire Grp3/4 cohort showing no array-based (i.e. 450k, black vs EPIC, grey) bias. **d.** t-SNE plot of entire Grp3/4 cohort shows no bias by source institution (Heidelberg, red; Newcastle, blue; Toronto, yellow).

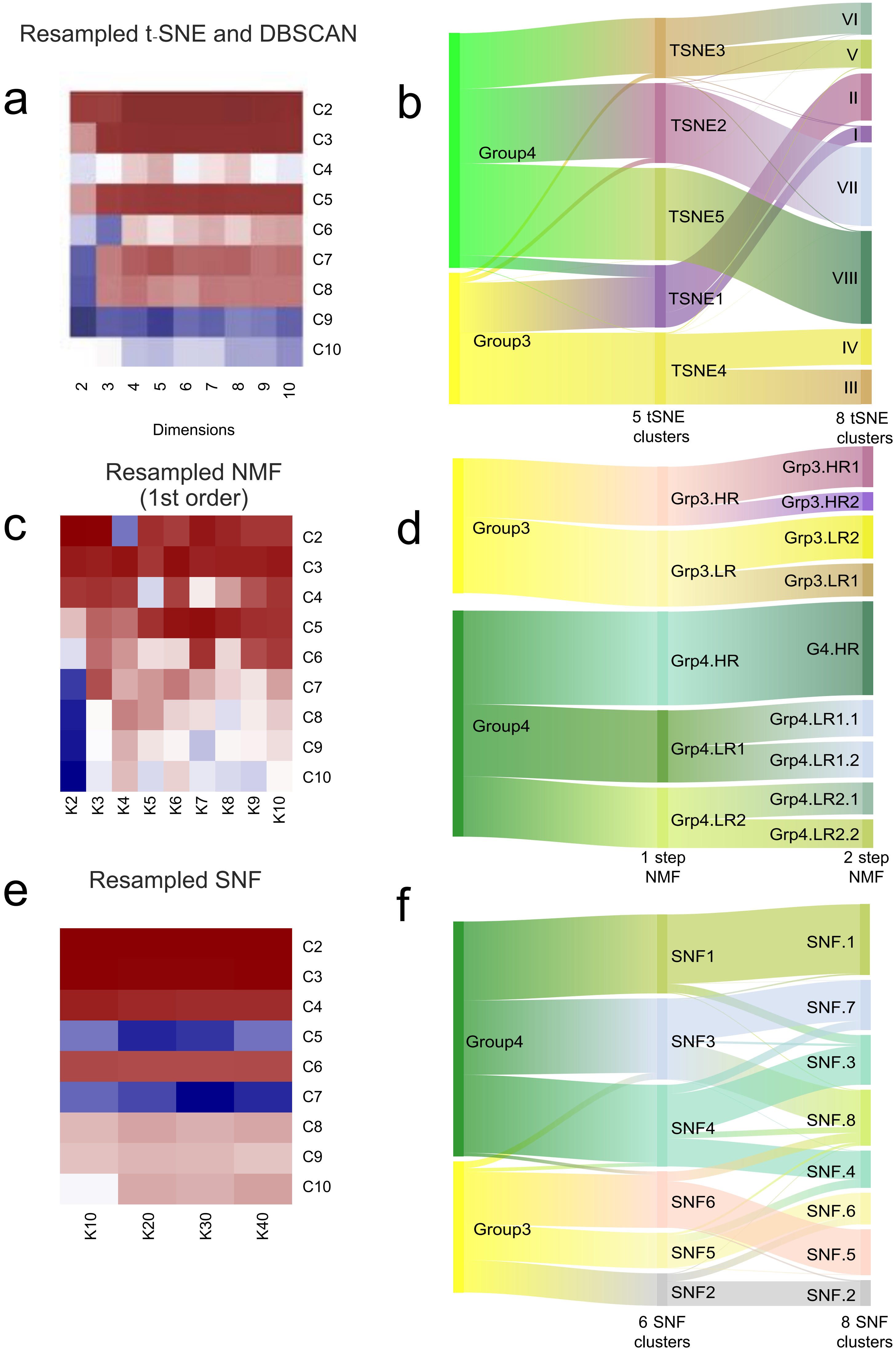

Resampled t-SNE and DBSCAN
a
C2
C3
C4
C5
C6
C7
C8
C9
C10
2
3
4
5
6
7
8
9
10
Dimensions
b
Group4
Group3
TSNE3
TSNE2
TSNE5
TSNE1
TSNE4
VI
V
II
I
VII
VIII
IV
III
5 tSNE clusters
8 tSNE clusters
Resampled NMF (1st order)
c
C2
C3
C4
C5
C6
C7
C8
C9
C10
K2
K3
K4
K5
K6
K7
K8
K9
K10
d
Group3
Group4
Grp3.HR
Grp3.LR
Grp4.HR
Grp4.LR1
Grp4.LR2
Grp3.HR1
Grp3.HR2
Grp3.LR2
Grp3.LR1
G4.HR
Grp4.LR1.1
Grp4.LR1.2
Grp4.LR2.1
Grp4.LR2.2
1 step NMF
2 step NMF
Resampled SNF
e
C2
C3
C4
C5
C6
C7
C8
C9
C10
K10
K20
K30
K40
f
Group4
Group3
SNF1
SNF3
SNF4
SNF6
SNF5
SNF2
SNF.1
SNF.7
SNF.3
SNF.8
SNF.4
SNF.6
SNF.5
SNF.2
6 SNF clusters
8 SNF clusters

Supplementary Figure 4. Selection of optimal subtype number through application of consensus clustering using t-SNE, NMF and SNF. a. Heatmap shows sample reproducibility for differing combinations of dimensions and resultant clusters using t-SNE. Maximal reproducibility was observed at 2 and 5 clusters. There was no support beyond a maximum of 8 clusters. **b.** Sankey plot shows relationship between Grp3/4, the 5 clusters and 8 clusters identified through consensus t-SNE clustering. **c.** Heatmap shows sample reproducibility for differing combinations of NMF-defined metagenes, denoted by K, and clusters, denoted by C. Maximal sample reproducibility was observed at 5 clusters, defined by 6 metagenes. Subsequently, 2nd-order NMF was performed individually on each of the five robust clusters identified to characterize any further subtype splits. **d.** Sankey plot shows relataionships between Grp3 and Grp4, the five clusters identified with 1st-order NMF and the additional subtypes identified with 2nd-order NMF. **e.** Heatmap shows sample reproducibility for SNF, with differing combinations of the k parameter and clusters, denoted by C. Maximal sample reproducibility was observed at 2 clusters, with a further local maximum at 6 clusters. Support for 8 stable clusters was also observed. **f.** Sankey plot shows relationships between Grp3 and Grp4, and for 6 and 8 clusters defined using SNF.

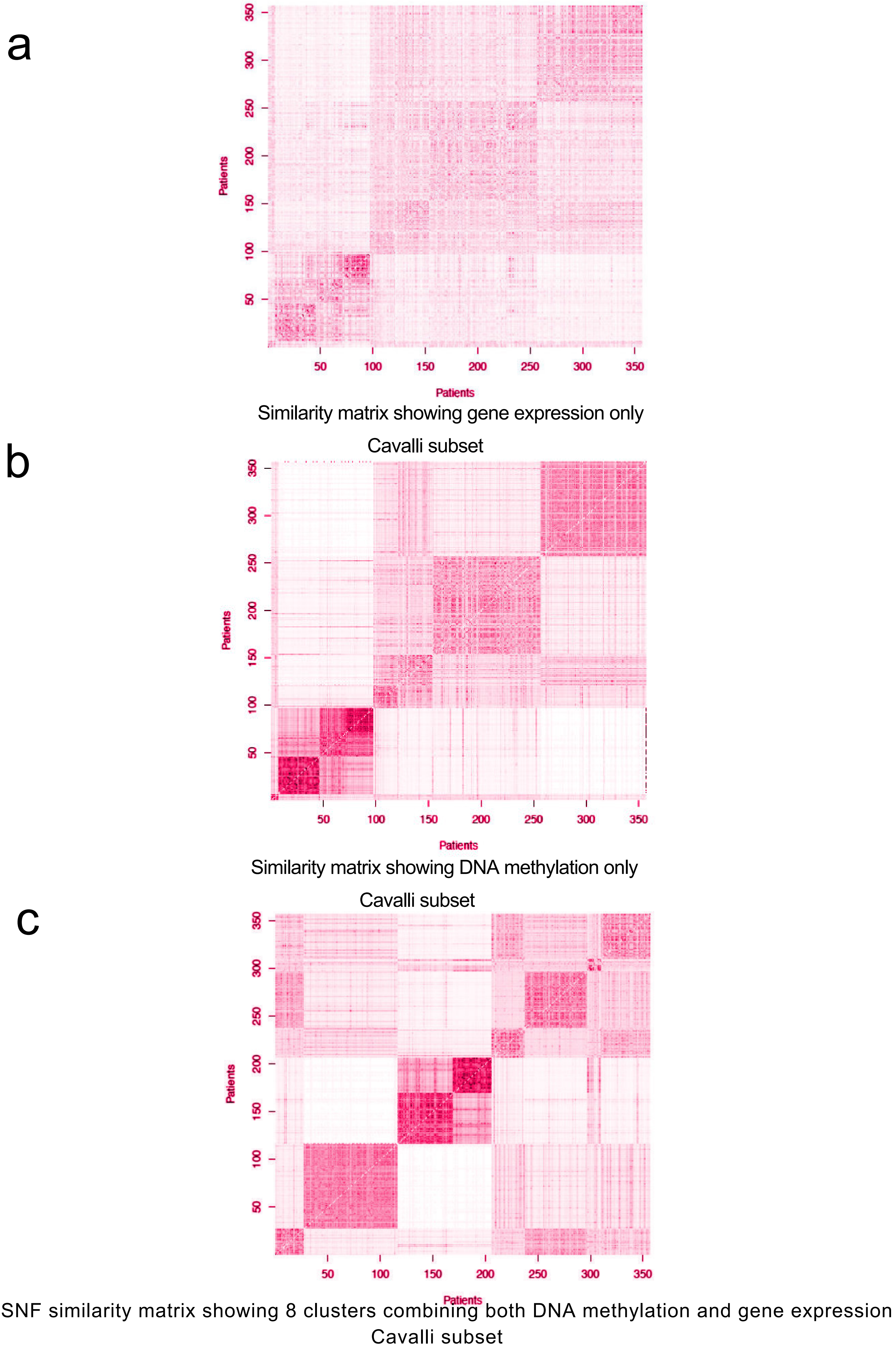
a
Patients
Patients
50
100
150
200
250
300
350
Similarity matrix showing gene expression only
Cavalli subset
b
Similarity matrix showing DNA methylation only
Cavalli subset
c
SNF similarity matrix showing 8 clusters combining both DNA methylation and gene expression
Cavalli subset

Supplementary Figure 5. Application of SNF clustering to the Cavalli et al dataset identifies eight subtypes. a. Similarity matrix for SNF performed using only gene expression data identifies 3 major subtypes, identified by the three major blocks. **b.** Similarity matrix for SNF performed on DNA methylation data identifies 5 clusters. **c.** Similarity matrix for SNF performed on paired DNA methylation and transcriptome data identifies eight subtypes.

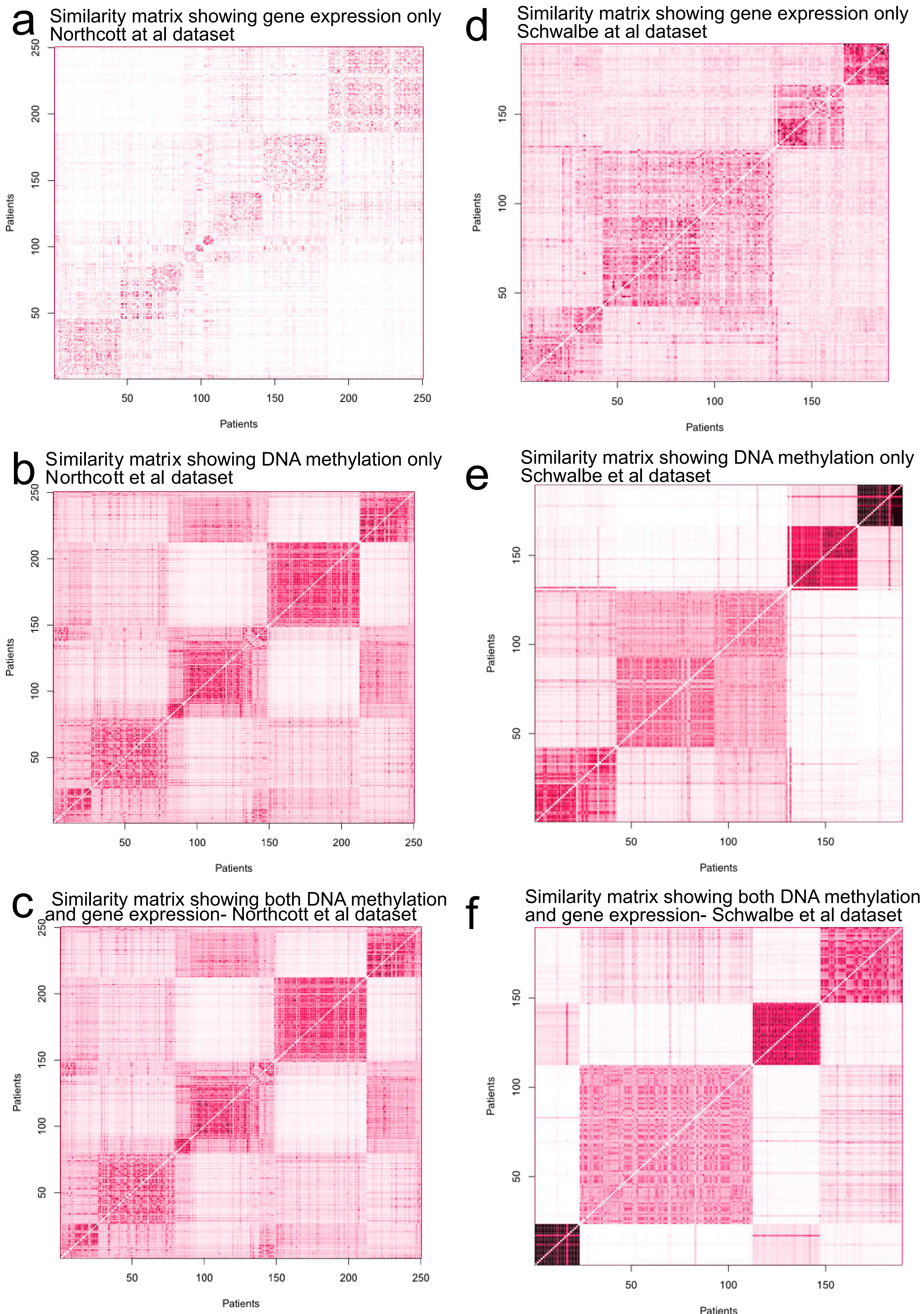
a
Similarity matrix showing gene expression only
Northcott at al dataset
b
Similarity matrix showing DNA methylation only
Northcott et al dataset
c
Similarity matrix showing both DNA methylation
and gene expression- Northcott et al dataset
d
Similarity matrix showing gene expression only
Schwalbe at al dataset
e
Similarity matrix showing DNA methylation only
Schwalbe et al dataset
f
Similarity matrix showing both DNA methylation
and gene expression- Schwalbe et al dataset
Patients
50
100
150
200
250

Supplementary Figure 6. SNF recapitulates study specific subtypes for Grp3/4 subsets of Schwalbe et al. dataset. a. The similarity matrix for SNF performed on RNA-seq data only identifies 4 subtypes for Schwalbe et al. dataset. **b.** The similarity matrix for SNF performed on DNA methylation data identifies 4/5 clusters. **c.** Stratification of the Schwalbe et al. dataset into 4 clusters using SNF matrix derived from combination of both DNA methylation and gene expression retrieves subtypes identified in the original study. **d.** Similarity matrix for SNF performed on gene expression data only on Northcott et al. subset fails to clearly stratify the data. **e.** Similarity matrix for SNF performed on DNA methylation data only on Northcott et al. subset identifies 5 clusters. **f.** Similarity matrix for SNF performed on paired DNA methylation and gene expression on Northcott et al. dataset identifies 5 clusters.

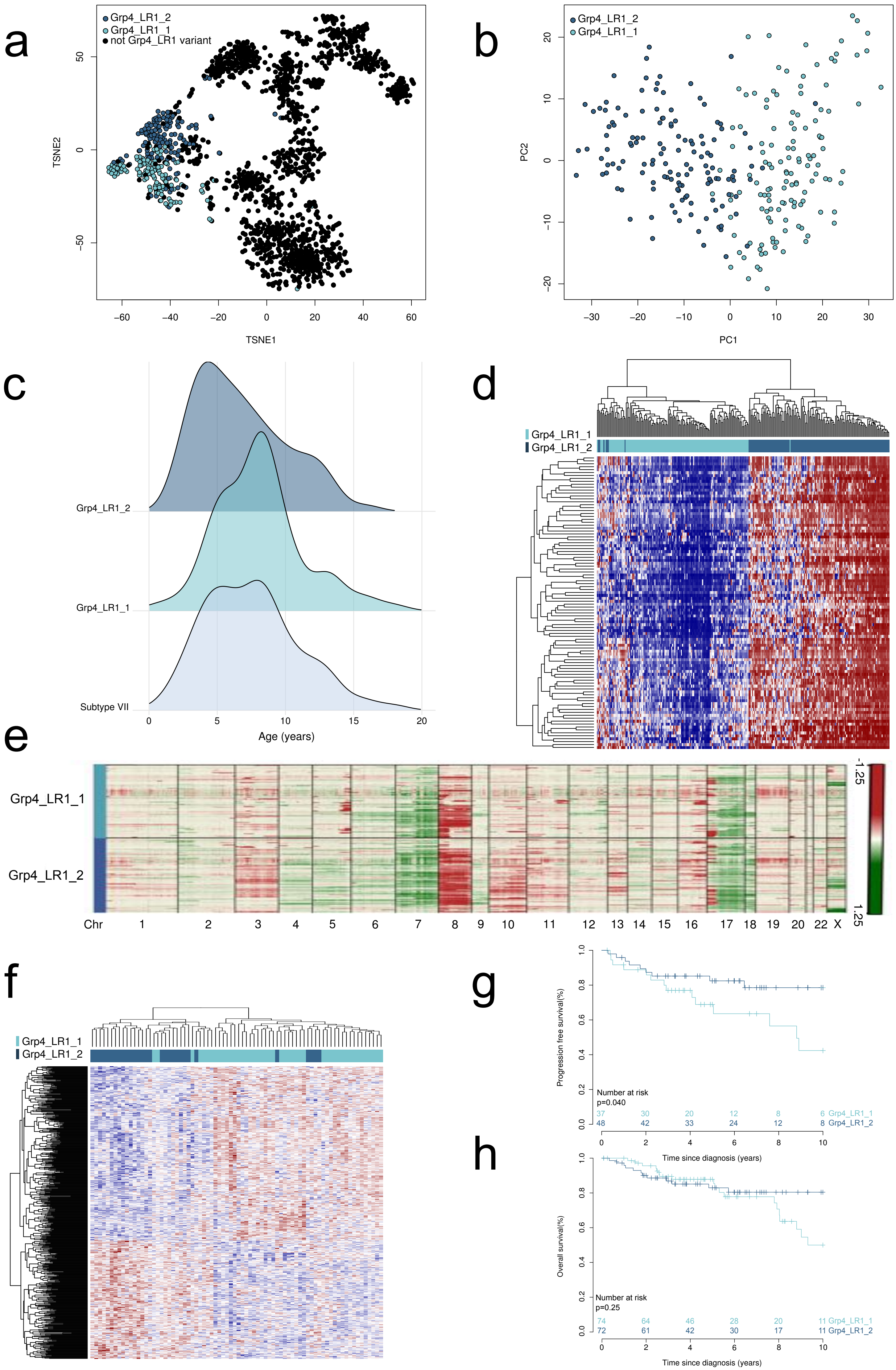

a
Grp4_LR1_2
Grp4_LR1_1
not Grp4_LR1 variant
TSNE1
TSNE2
b
Grp4_LR1_2
Grp4_LR1_1
PC1
PC2
c
Grp4_LR1_2
Grp4_LR1_1
Subtype VII
Age (years)
d
Grp4_LR1_1
Grp4_LR1_2
e
Grp4_LR1_1
Grp4_LR1_2
Chr
-1.25
1.25
f
Grp4_LR1_1
Grp4_LR1_2
g
Progression free survival(%)
Number at risk
p=0.040
37 30 20 12 8 6 Grp4_LR1_1
48 42 33 24 12 8 Grp4_LR1_2
Time since diagnosis (years)
h
Overall survival(%)
Number at risk
p=0.25
74 64 46 28 20 11 Grp4_LR1_1
72 61 42 30 17 11 Grp4_LR1_2
Time since diagnosis (years)

Supplementary Figure 7. NMF identifies two variants of subtype VII. a. t-SNE plot of entire Grp3/ Grp4 cohort, showing two subtype VII variants (Grp4_LR1_1, light blue; Grp4_LR1_2, dark blue; other samples, black). **b.** PCA plot of subtype VII NMF-variants only, shows separation of variants. **c.** Age distributions of subtype VII, Grp4_LR1_1 and Grp4_LR2_2. **d.** Heatmap shows most variably methylated loci that distinguish variants. Unmethylated loci are shown blue, methylated are shown red. **e.** Copy number differences between NMF subtype VII variants. **f.** Gene expression differences between NMF-derived subtype VII variants for Cavalli et al. transcriptome dataset. **g, h.** Progression-free and overall survival plots for subtype VII variants Grp4_LR1_1 and Grp4_LR2_2.

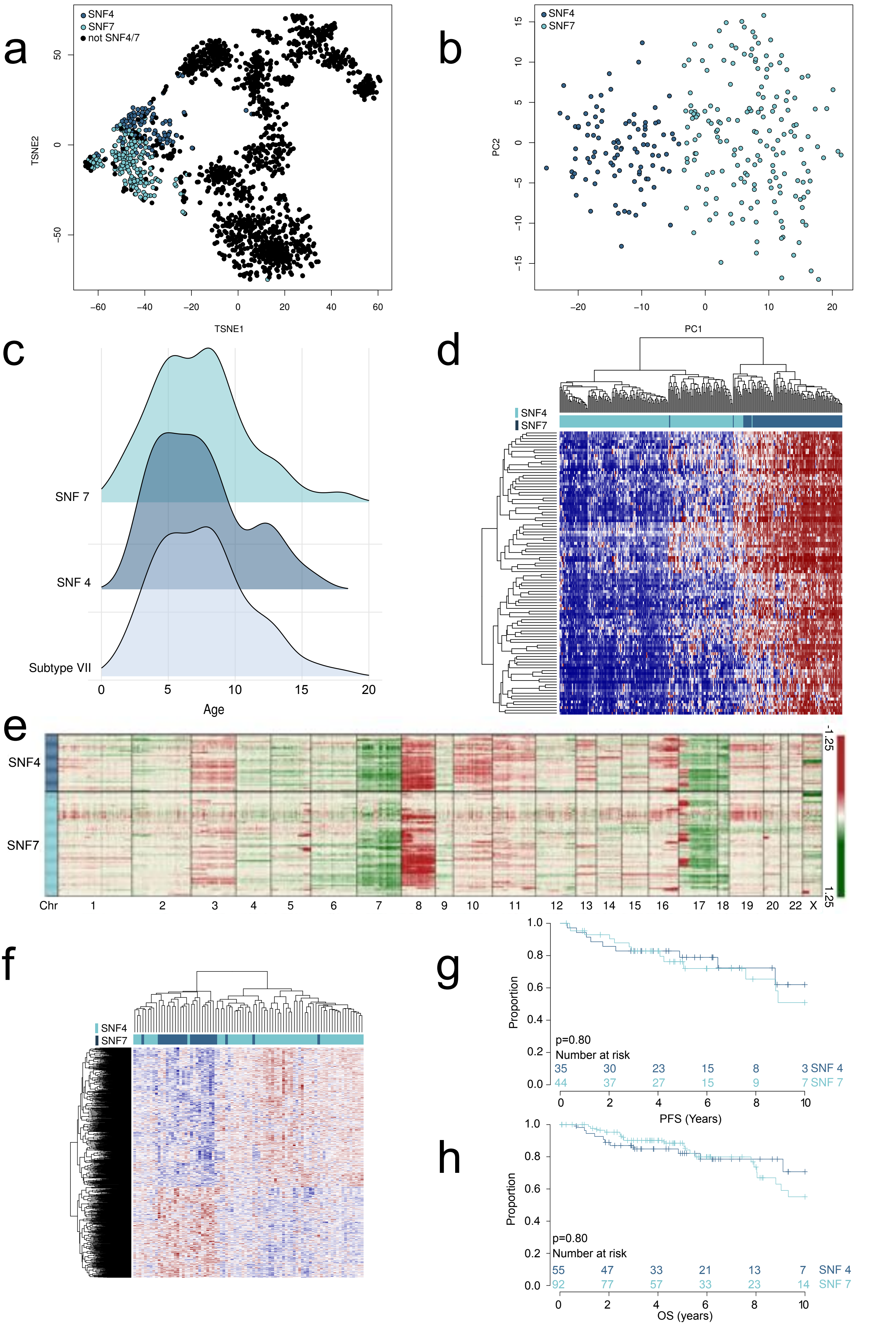

a
SNF4
SNF7
not SNF4/7
TSNE1
TSNE2
b
SNF4
SNF7
PC1
PC2
c
SNF 7
SNF 4
Subtype VII
Age
d
SNF4
SNF7
e
SNF4
SNF7
Chr
-1.25
1.25
f
SNF4
SNF7
g
p=0.80
Number at risk
35 30 23 15 8 3 SNF 4
44 37 27 15 9 7 SNF 7
Proportion
PFS (Years)
h
p=0.80
Number at risk
55 47 33 21 13 7 SNF 4
92 77 57 33 23 14 SNF 7
Proportion
OS (years)

Supplementary Figure 8. SNF identifies two variants of subtype VII. a. t-SNE plot of entire Grp3/ Grp4 cohort, showing two subtype VII variants (SNF 7, light blue; SNF4, dark blue; other samples, black). **b.** PCA plot of subtype VII SNF-variants only, showing separation of variants. **c.** Age distributions of subtype VII, SNF4 and SNF7. **d.** Heatmap shows most variably methylated loci. Unmethylated loci are shown blue, methylated are shown red. **e.** Copy number differences between subtype VII variants. **f.** Gene expression differences between SNF-derived subtype VII variants using Cavalli et al. transcriptome dataset. **g, h.** Progression-free and overall survival plots for subtype VII variants SNF4 and SNF7.

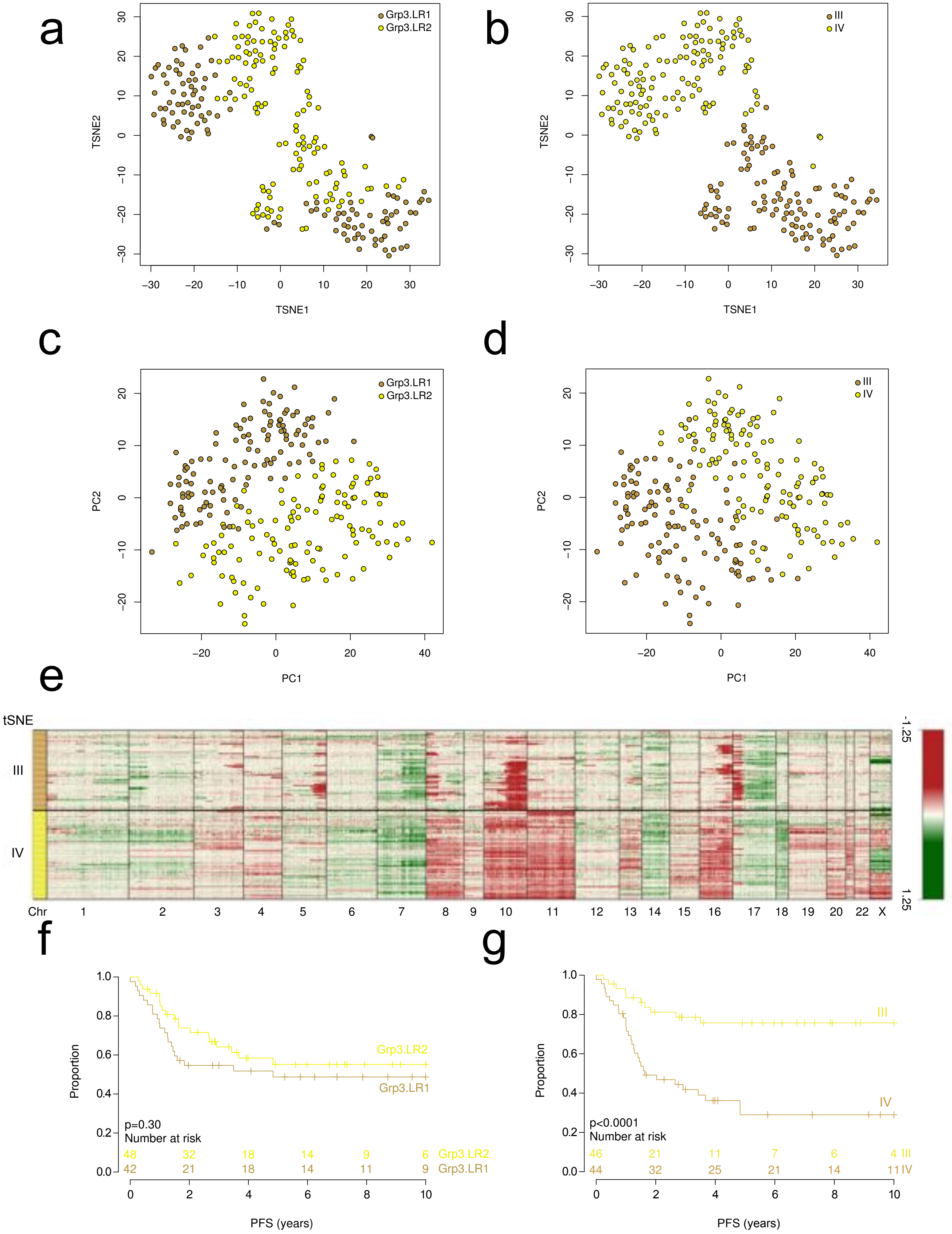

a
Grp3.LR1
Grp3.LR2
TSNE2
TSNE1
b
III
IV
TSNE2
TSNE1
c
Grp3.LR1
Grp3.LR2
PC2
PC1
d
III
IV
PC2
PC1
e
tSNE
III
IV
Chr
1 2 3 4 5 6 7 8 9 10 11 12 13 14 15 16 17 18 19 20 22 X
-1.25
1.25
f
Grp3.LR2
Grp3.LR1
Proportion
p=0.30
Number at risk
48 32 18 14 9 6 Grp3.LR2
42 21 18 14 11 9 Grp3.LR1
PFS (years)
g
III
IV
Proportion
p<0.0001
Number at risk
46 21 11 7 6 4 III
44 32 25 21 14 11 IV
PFS (years)

Supplementary Figure 9. NMF incorrectly partitions consensus subtypes III/IV. a,b. t-SNE plot of subtypes III and IV labelled (**a**) by NMF-derived subtypes, and (**b**) by t-SNE-derived subtypes. **c, d.** PCA plot of subtypes III and IV labelled by (**c**) NMF-derived subtypes, and (**d**) t-SNE-derived subtypes. **e.** Distinct copy-number differences are observed for III/IV subtypes that were not identified in the NMF-derived subtypes. **f, g.** Progression-free survival plots for (**f**), NMF-derived subtypes, and (**g**), t-SNE-derived subtypes support the adoption of III/IV subtypes in favor of NMF-derived subtypes.

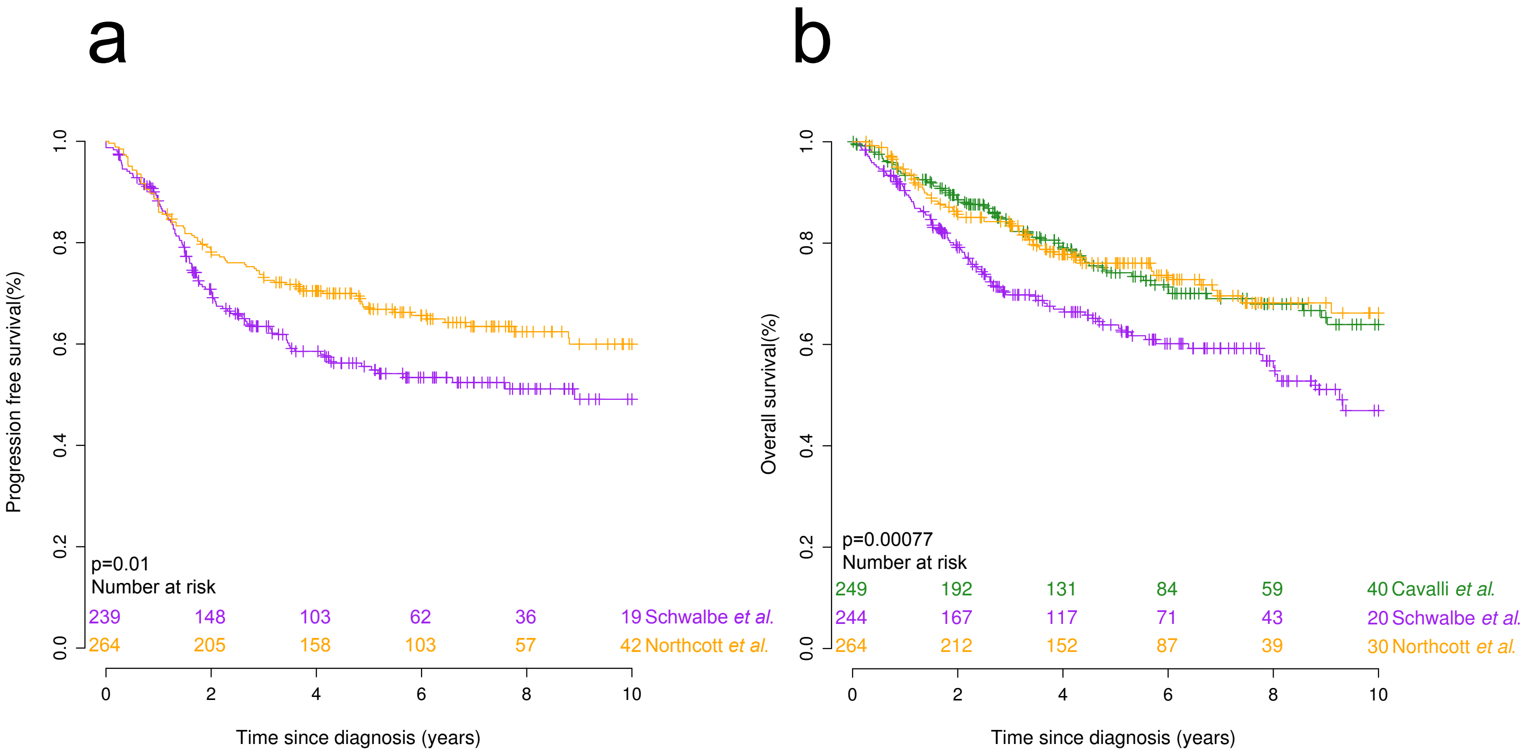

Supplementary Figure 10: Survival analysis identifies cohort-specific survival differences. a. Kaplan-Meier plot shows progression free survival for Schwalbe *et al.* and Northcott *et al.* cohorts. **b.** Kapan-Meier plot shows overall survival for Schwalbe *et al.*, Cavalli *et al.* and Northcott *et al.* cohorts. At risk tables are shown in two-year increments.

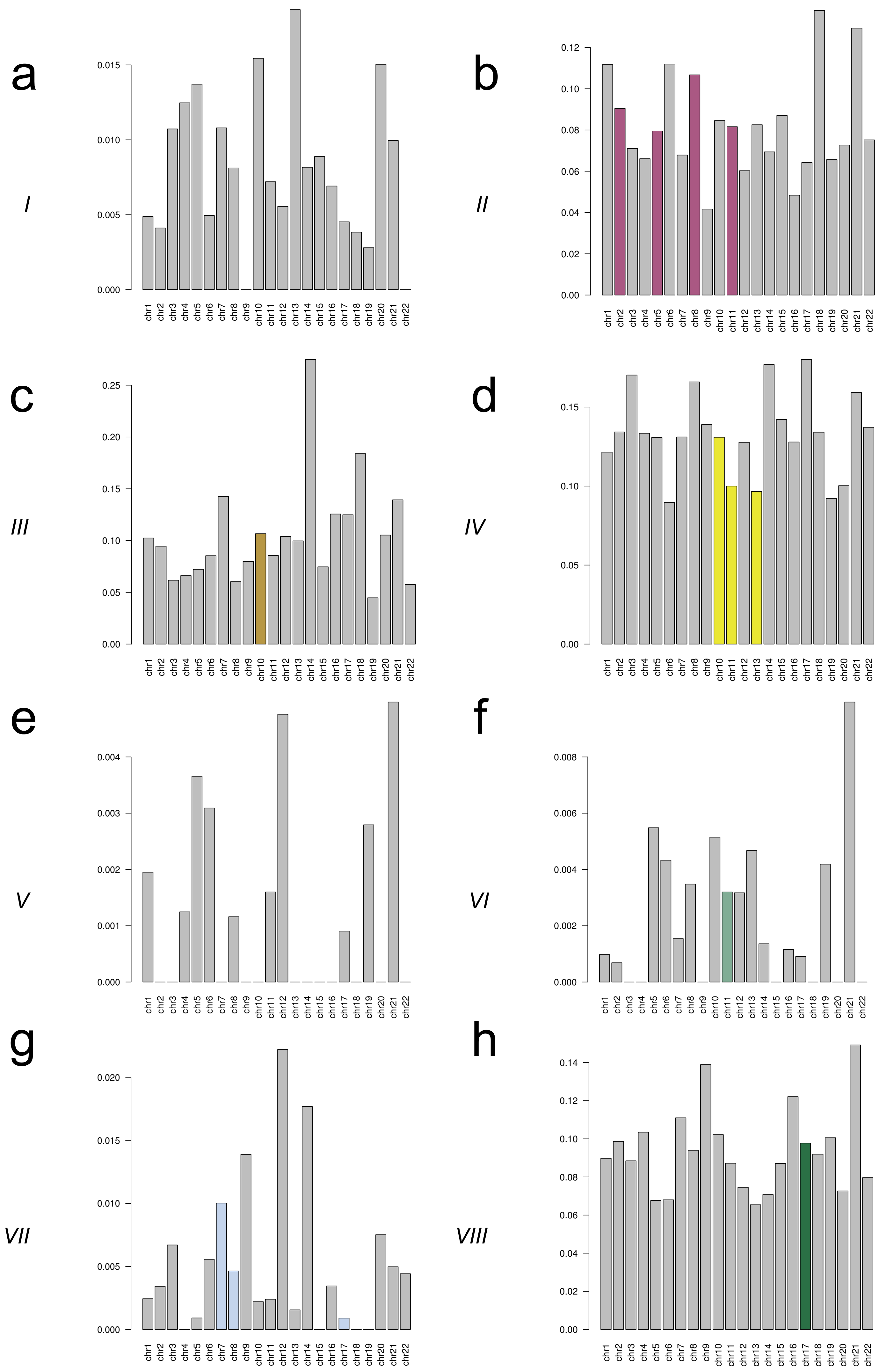
a
I
0.000
0.005
0.010
0.015
b
II
0.00
0.02
0.04
0.06
0.08
0.10
0.12
c
III
0.00
0.05
0.10
0.15
0.20
0.25
d
IV
0.00
0.05
0.10
0.15
e
V
0.000
0.001
0.002
0.003
0.004
f
VI
0.000
0.002
0.004
0.006
0.008
g
VII
0.000
0.005
0.010
0.015
0.020
h
VIII
0.00
0.02
0.04
0.06
0.08
0.10
0.12
0.14
chr1 chr2 chr3 chr4 chr5 chr6 chr7 chr8 chr9 chr10 chr11 chr12 chr13 chr14 chr15 chr16 chr17 chr18 chr19 chr20 chr21 chr22

Supplementary Figure 11: Subtype-specific differentially methylated loci are not enriched in corresponding significantly altered chromosomes. For each subtype I-VIII, the chromosomal distribution of differentially methylated loci (mean change in beta value >0.3, qval < 0.0001) is shown, relative to the overall number of testable CpG loci per chromosome. Significant, subtype-specific chromosomal alterations (i.e. gain/loss) are shown in the corresponding subtype colour. Chromosomes without significant copy number alterations in a subtype are shown grey.
